# Leveraging innovative technologies for designing a healthy and personalized breakfast: consumer perception of three smart cooking devices in the EU

**DOI:** 10.12688/openreseurope.14234.3

**Published:** 2022-05-03

**Authors:** Clara Talens, Yolanda Rios, Elena Santa Cruz

**Affiliations:** 1 New Foods, AZTI, Food Research, Basque Research and Technology Alliance (BRTA), Derio, Bizkaia, 48160, Spain

**Keywords:** consumer perception, innovation, technology, 3D food printing, personalized nutrition

## Abstract

Background: Rapid demographic growth and global population ageing will have profound impacts on food and nutrition. In the long run, the smart appliance industry will reflect the social, technological, and demographic forces around food consumption. This study aims to provide valuable consumer insights about smart home cooking devices for a healthy and personalised breakfast.

Methods: Three smart cooking technologies (3D food printing, digital sous-vide cooking and instant dough baking), one ingredient supplier, and a food research centre shared resources to carry out common tasks such as market research and consumer validation tests. Consumers were segmented into four types of households (single, young or consolidated families and senior). An online community (40 participants), nine focus groups, two interviews (8 experts), and one quantitative study (2055 participants) were carried out in Spain, UK, and Germany.

Results: Consumer perception of three smart cooking devices in the EU has been explored in-depth. Insights have shown the potential of innovative technologies for designing a healthy and personalised breakfast. A customized, fresh, tasty, nutritious, and healthy 3D printed breakfast bar was developed for senior consumers by using the smart cooking devices. A tasting session with 80 senior Spanish consumers, aged 45 to 75 years, showed that around 56% of consumers increased their acceptance of the new breakfast bar after being informed about its technological, nutritional, and convenient benefits.

Conclusions: The findings provided both theoretical and practical insights into the perception of the three smart cooking devices, per type of household and per country. A combination of technologies was used to develop the new breakfast concept for the target group and country with the most positive perception. This study shows how to share resources for gathering information on product attributes, consumer experience, and for validating the new concept with the target group identified via consumer market research.

## Plain language summary

Imagine the kitchen of the future, including a new generation of smart kitchen appliances, interconnected and connecting people via the internet, preparing your customized, fresh, tasty, nutritious and healthy breakfast, according to your instructions or preconfigured recipes. In the meantime, you are taking a shower, waking up the kids or just relaxing checking your social networks or chatting with your friends.

As suggested by nutritionists, breakfast may vary between life stages, but in general breakfast is essential to get the “first shot” of energy for the day. Nowadays, consumers have limited time for breakfast. In addition to fast and healthy, the current solutions for fast breakfast (many prepacked products) are not giving the pleasure of fresh food. The flexible usage of smart appliances connected to the internet, and the combination of nutritious ingredients, will enable fulfilment of the most important meal of the day.

In this research, three different prototypes of smart cooking devices were used for the preparation of a customized, fresh, tasty, nutritious, and healthy breakfast, prepared in a convenient way. This work analysed what consumers perceived about breakfast and about new smart cooking devices designed to prepare it, in four different types of households: single (from 20 to 35 years old, living alone, shared flats and/or young couple without children), young families (from 30 to 45 years old, single-parent or nuclear families, with children from zero to seven years old), consolidated families (from 46 to 60 years old, with at least one child living at home aged between 8 and 25 years old) and senior from 60 years old, onwards, living alone or parents without children at home or with children older than 25 years old); and in three countries, representing three different types of traditional breakfast: Spain (Mediterranean), Germany (Continental) and UK (English).

## Introduction

Rapid population growth, increasing urbanization, and expansion in the global middle class will profoundly affect the quality of food and nutrition ( Wells & Stock, 2020). It is to be expected that the future smart-home appliance industry will be shaped by the social, technological, and demographic environment without losing the authenticity of food in the kitchen ( Kim *et al*., 2019). Cutting-edge kitchen appliances will collaborate with their human owners in planning and shopping for meals, helping to fulfil specific dietary needs, and creating improved lifestyles. New consumer-centric solutions will result in innovative, customized food products within the next few decades ( Stander *et al*., 2012). Even though most of the product development and consumer research has been initiated by the launch of home beverage appliances ( Brem *et al*., 2016), many innovative platforms are still emerging. This has presented new challenges in assessing the relevant attributes of these platforms. The existing data indicate some limitations in the strategy of consumer-centric product launches; however, the recent developments open the way for exploring new experiences and attributes sought by the consumers. Overall, the technology helps consumers make informed, personalized nutrition choices using direct engagement, giving them access to new product concepts and informative packaging. The use of appliances is driven mainly by convenience; therefore, increasing or upgrading appliance capabilities based on consumer feedback will improve the interaction and engagement of consumers.

In particular, among elderly people, a healthy breakfast is important for maintaining their health and quality of life. To fill the gap in the range of appropriately nutritional breakfast products, the search for alternative highly nutritional sources and the development of new products with such ingredients must be conducted ( Bell *et al*., 2020). In the elderly care sector, the appearance of the food served can be improved by thickening and shaping using moulds, which is a time-consuming process. This presents an additional task for the staff; every measure to pre-prepare or pre-process food for the residents results in labour-intensive processes to be performed by the already stretched staff. Thus, such care facilities are actively looking for solutions to produce acceptable nutritional meals using a less labour-intensive or, ideally, partially automated process.

The SmartBreakfast project, a part of the EIT Food innovation program, combines several prototypes of smart appliances to prepare a tasty, nutritious, convenient, and personalized breakfast. It monitors critical nutrients (sugar, salt, fat, etc.) to raise consumer awareness and control unhealthy nutritional habits. The main actors taking part in the project were three start-ups ^1^, with a 3D-food printer, an instant dough baking device, and a *sous-vide* cooking device at their disposal; a top-tier food research innovation centre ( AZTI, Spain) and an industrial ingredient supplier ( Puratos NV, Belgium) were also involved.

For the success of start-up companies who are often developing new technologies, the strategic collaborative relationships with suppliers and customers are vitally important. Such relationships complement their capabilities and help make efficient use of their scarce resources ( Laage-Hellman *et al*., 2018). Collaboration with different partners can significantly reduce innovation costs and risks. The customers are the best source of information on the desirability of the product that a start-up should produce.

This work provides insights into how resources for common tasks such as market research, product development and consumer taste tests can be shared among the actors in this food context (environment) (three start-ups, one top-tier food research and innovation centre and an industrial ingredient supplier) for a common goal: to obtain information capable of contributing to the co-creation of personalized food for different consumer groups: 46–60 years (mature) and 61–75 (senior).

The countries selected for the study were Spain, UK, and Germany because of their cultural differences between their breakfast habits: Mediterranean breakfast (Spain), Central-European breakfast (Germany) and English breakfast (UK). Traditionally, a Mediterranean breakfast includes products such as coffee, milk, bakery products, bread, ham, olive oil and fruit; continental breakfast includes products such as tea or coffee, citrus fruits, scrambled eggs, ham, cheese, butter, cured meat and wholemeal bread; and an English breakfast can include tea, yoghurt, berries, oats, butter, jam, fried eggs, beans, bacon, black pudding, and toasted bread.

The purpose of this study is to provide valuable consumer insights about three smart home cooking devices (3D food printer, digital sous-vide cooker and instant dough baker) for designing a healthy and personalised breakfast, in 4 type of households (single, young families, consolidated families and senior); and in three countries, representing three different types of traditional breakfast: Spain (Mediterranean), Germany (Continental) and UK (English).

## Methods

A combination of methodologies was used: two qualitative studies (an online community and nine face-to-face focus group sessions) with the aim of evaluating the potential of the technologies; and one quantitative study: a questionnaire formulated to determine the perception of the different devices among potential consumers.

The methodology used in this study covers two of the most common needs among start-ups: market research and formula validation. By collaborating and sharing resources more efficiently, the start-ups, supported by the European Institute of Innovation & Technology (EIT Food), will aim to improve their market uptake by analysing product experiences-attributes and validation through consumer market research of smart appliances and the human-device interaction during breakfast. This methodology involved consumers as well as stakeholders (retailers, Horeca) managers and chefs), on the design and perception of new food products using the smart cooking devices (see in Data file 2).

The 3D-food printer prototype can print a wide range of foods, both savoury and sweet, and uses up to five open capsules that are automatically exchanged as needed. The printer uses an open-capsule model; the consumer prepares and places fresh ingredients in the capsules. The capsules are printed one at a time; each has its own food-grade twist-off nozzle. The nozzle deposits exact fractional layers directly onto a plate or other surface in a layer-by-layer additive manner (please see Data file 2 and 3 in Extended Data).

The *sous-vide* cooking prototype device is a smart cooking appliance that can be remotely monitored from its mobile application. Also known as low-temperature long-time cooking, the *sous-vide* is a cooking method in which the food is placed in a plastic pouch or a glass jar and cooked in a water bath: cooking times are longer and the temperature lower than in conventional cooking. The user has access to a wide range of *sous-vide* recipes in the mobile application. The device maintains the constant temperature of the water bath during the appropriate time.

The instant dough baking prototype uses the capsules to make flatbreads using different food ingredients with different flavours; the flatbread can be combined with other breakfast elements.

The three start-ups provided the smart cooking devices for the study, the top-tier food research and innovation centre (AZTI) provided know-how on formula development and nutritional claims, consumer perception and industrial scale-up. Finally, the industrial ingredient supplier (Puratos) supplied bakery ingredients and mixes.

### Market research techniques for common targets

Three different methodologies were used for gathering information on product experiences and attributes, and validation through consumer market research of smart appliances and their interactions during breakfast.

The objectives were to (1) conduct a market segmentation via an online community to identify the most interested target audience for each device per country, (2) to carry out a qualitative research analysis (offline demonstration) via focus groups, to go into detail about the usability and consumer experience of each target audience more interested in each device per country; and (3) to statistically represent the opinions provided by the participants of the online community and the nine focus groups, in order to quantify the market opportunity for each device in each country. Participants were recruited from a marketing agency database using purposive sampling. For those who expressed an interest to participate, an email was then sent to confirm their interest, attaching the participant information sheet explaining the purpose of the study and the monetary compensation. Qualitative researchers with vast experience in market and social research carried out the study using ethnographic methods. Their relationship with participants was only for the purpose of the study. The participants did not know the researchers and all of them completed the study. In the different stages of the research, the sample composition was designed with a balanced representation of the three countries, the four types of households, gender, education and social status ( Table 1).

**Table 1.** Socio-demographic characteristics (in %) of the online survey (n = 2055).

countries	
Spain	38.4
Germany	36.6
United Kingdom	25.0
**gender**	
male	59.2
female	40.8
**children**	
yes	55.5
no	44.5
household composition	
single	49.5
young families	24.3
consolidates families	11.0
senior	15.1
age	
20–29	32.5
30–35	34.5
36–45	8.9
46–60	9.1
61–75	15.1
education	
illiterate	0.6
primary	3.2
secondary	45.0
university	51.2
occupation	
working/employed	74.1
unemployed	3.0
houseworker	4.8
student	7.3
retired/pre-retired	10.0
unable to work	0.7
social status *	
upper	8.4
upper-middle	20.3
middle class	46.0
lower-middle	22.9
low	2.3

*Participants self-classified themselves into a social class status on the basis of their own self-assessment and income. ( *Could you please tell me to which social status you and your family belon* *g*?).

All participants granted written informed consent prior to participating in the survey. The ethical approval was conducted according to quality standards of the ISO 20252 certified by AENOR, the Spanish Association for Standardization and Certification and the ICC/ESOMAR International Code on Market and Social Research ( European Society for Opinion and Marketing Research, ESOMAR, 2006). For the online survey, guidance was provided by the interviewer, and pilot testing was carried out with a smaller sample before launching=. Materials for the focus groups and on-line community were also pilot tested internally before being launched. The audio was recorded, and notes were filed during the process. Data saturation was not discussed, and transcripts were not returned to participants for comment or correction.

**Figure 1.**
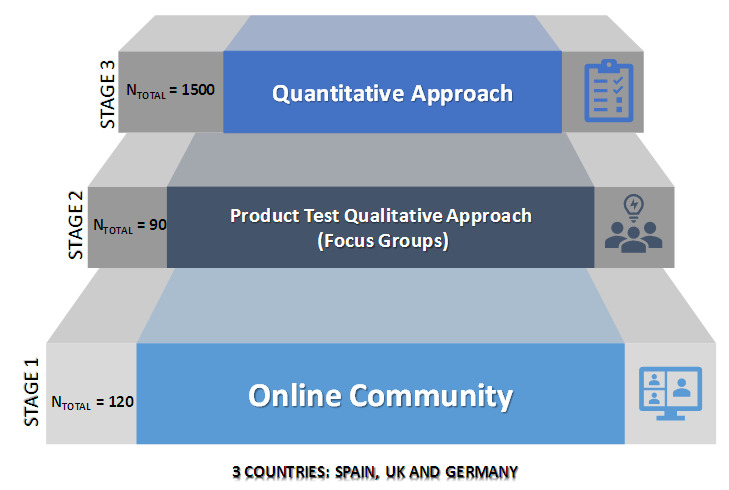
Stages and methodologies followed during the research.

As a first stage of the research ( Figure 1), a private online community was created for a three-month study using a multi-channel tool to identify the breakfast habits and preferences (questions such as what, why, where, how, preferences) of the target group most interested in each device, in each country. The multi-channel tool optimized the opportunities to communicate with potential customers across different web platforms and channels.

The online community has been revealed as being an advantage to obtain valuable insights from consumers ( Hunter & Stockdale, 2010) with specific expertise, preferences, needs or habits. The community consisted of 40 participants (10 x 4 types of household) from each country (a total of 120 individuals, from 15 to 60 years old). The type of households recruited are described in Table 2.

**Table 2.** Description of the four types of households used for the study.

**Single** • Alone • Shared flats • Young without children • From 20 to 35 years old	**Young families** • Single-parent families • Nuclear families • Children from 0 to 7 years old • From 30 to 45 years old
**Consolidated families** • Single-parent families • Nuclear families • Children from 8 to 25 years old • At least one child living at home • From 46 to 60 years old	**Seniors** • Alone • Without children at home • With adult children at home > 25 years. • From 60 years old, onwards

Participants were invited to answer mini-surveys and longer surveys, and actively take part in forums and group discussions, being able also to upload audio-visual materials. Members became involved and remained within the online community because of the reciprocal exchange of information regarding the topics and the smart cooking devices presented. 40 participants were recruited in each country (Spain, UK and Germany). Moderators were previously briefed and followed a detailed discussion guide (see Data file 1 in Extended Data). The main objectives were to gather insights on habits and preferences on breakfast, to explore consumer perception of the three smart cooking devices and to identify the target audience who were most interested in each device per country. An exploratory qualitative analysis was conducted in order to investigate the type of answer and posts published in the platform as well as the characteristics and emotions transmitted through them ( Gheorghe & Liao, 2012). Content analysis, which is a common method for analysing messages on online forums ( Gremler, 2004), was used to analyse the postings.

The questions were asked during seven independent forums. The questions used are provided in the extended data (Data file 1). All questions listed were asked to the online community and participants were invited to respond.

In a second stage, nine focus groups were held as another qualitative approach to gain a deeper understanding of consumers’ habits and preferences about breakfast, as well as their perceptions and opinions about the smart cooking devices. This is an effective product marketing research tool as it allows examination of everyday life as well as a deeper understanding that explains consumers' opinions and assessments of the proposed new developments. The focus groups were conducted in six cities from the targeted countries: Spain (Madrid, Barcelona, Bilbao), Germany (Munich, Berlin), UK (London). Consumers were recruited from the online community and were invited to participate in the offline demonstration (via focus groups). Each focus group consisted of 25–27 participants belonging to the type of household with the highest rate per each device, per country. They were conducted in professional research facilities. Each focus group lasted two hours approximately, and between eight and ten people were recruited. The moderators were previously briefed and followed a detailed discussion guide that included the following sections: habits and preferences for breakfast, product perception, attributes and recommendations. Participants were asked to evaluate the breakfast bar and decide the potential market success of the products as they were frequent users of smart cooking devices. Results were analysed in terms of spontaneous reactions, prompted reactions, featured values, targets, place of purchase and projected future. The above-described focus group sessions were conducted in the native language of each country (Spanish, German, and English), audio recorded and videotaped, simultaneously translated into English, and verbatim-transcribed for further traditional semantic analysis from the English translation to draw the main conclusions ( Stewart *et al*., 2007).

A quantitative study (third stage) was also conducted to provide scientific and objective knowledge to guide the commercial strategy and market development according to targets, devices and countries. The first objective was to do an in-depth study of the relevance of the different meals of the day, with a special focus on breakfast, nutrients desired, places of consumption and use of appliances. For this purpose, a questionnaire was designed using the results, experiences and learnings of the online community and the focus groups. The second objective was to statistically represent the opinions provided to quantify the market opportunity for each device in each country.

Using the qualitative test results, four online questionnaires were designed. To avoid comparison among devices, the first three questionnaires focused on just one device only. The fourth one was designed for comparing the three devices together. The intention was to assess the effect of the other two devices on the evaluation of each device by the consumer. Individuals were randomly selected for each questionnaire in order to have a balanced sample for each device.

The universe of study was the population responsible for food product purchases in their household in Spain, Germany and UK, aged between 20 and 75, representing 2055 participants in total. Participants matching the profile required were recruited via a consumer panel in each country, and the sampling error was ± 4.42 % for the total sample, with a confidence level of 95.5 %. The sample distribution was by country and type of households ( Table 2): 685 participants in Spain, 685 in Germany and 685 participants in UK. Results were obtained through an online semi-structured survey (CAWI System, Survey Solutions version 5.25), with an average duration of 30 minutes. The link to the survey was shared with participants via email by the consumer panel platform.

### Interviews with experts

To explore the commercialization synergies between start-ups, mean scores for each type of household were calculated. The type of household with the highest score was selected as the target group. The size of the sample was decided as per Guest *et al*. (2006). In depth interviews were developed in Spain and Germany in order to check the suitability of the designed methodologies and the user experience and market potential of the smart breakfast technologies developed in the project. As their participation was voluntary, interviews with eight experts from the hospitality and service sector were carried out in those countries, with the following expertise: restaurant management (three in Spain), geriatric care management (two in Germany), catering management (one in Spain), and culinary arts education (two in Spain).

### Development of nutritionally balanced formulae for "SmartBreakfast."

After obtaining feedback on the most common breakfast dishes, targeted formulations were developed for each device.

All the formulations contained nutritious and sustainable ingredients, and the recipes were designed to produce the different breakfast bar components, using the three smart appliances.

The design and development of breakfast formulae were carried out using industrial ingredient suppliers to facilitate the scale-up of the recipes in the future. All the ingredients came with their corresponding technical sheets; the data were used to estimate the nutritional profile of the formulae before selecting the final prototypes. For example, the selection of the final prototypes for the 3D food printer was based firstly on the texture and flowability in printing, secondly on the sensory properties assessed internally, and finally, on the estimated nutritional profile. The nutritional profiles of the selected formulae were analysed following the Association of Official Agricultural Chemists (AOAC), 2016 standard procedures for sugar content, ashes, dietary fibre, moisture content, carbohydrates, sugar, protein, insoluble dietary fibre, fat, saturated fat, sodium, salt (from sodium), caloric value and fatty acids profile (by chromatography). To assess the possible nutritional claims, the Regulation (EC) No 1924/2006 of the European Parliament and the Council of 20 December 2006 on nutrition and health claims made for foods was followed.

The sensory properties and acceptability of the selected prototype were then assessed by the targeted group defined in the market research. The study was carried out using a quantitative test with a structured scale of nine points (according to the UNE-ISO 4121: 2006 standard), grading the appearance, smell, taste, texture, and overall impression; ranging from 1 = *I dislike extremely* and 9 = *I like extremely*. For the acceptability test, 80 senior consumers aged between 45 and 75 years, 65% women and 35% men, all of them residents in the Biscay region (Spain) were recruited. After explaining the product concept to consumers (personalized breakfast bar, nutritious, with fresh ingredients and ready to go), they were asked how they thought it was going to taste. Afterwards, to the concept of "personalized breakfast bar, nutritious, with fresh ingredients and ready to go", the attribute “freshly made” was added. Then, the product was presented to the consumer. So, first expectation was assessed, and then acceptability.

### Data analysis

For the statistical analysis of the results, R-project software (version 3.4.3) was used. Simple and cross tabulation of frequencies were performed segmented by country and device. Chi-squared test was performed on frequency data and effects showing a p-value of 0.05 or lower were considered significant.

## Results

### Consumer feedback and its role in food product development

Results of the three-month online community test are explained in terms of meanings of breakfast, places where to have breakfast, origin of products consumed at breakfast, feelings linked to breakfast, the ideal breakfast, appliances used, special foods and healthy breakfast perception.

### Meanings of breakfast

In general, participants highlight the functional aspect of breakfast, being its abundance and variety the most valued aspects (3.95/5). Note that health and nutrition at breakfast were ranked as the lowest relevant aspects in all the different types of households -both in Germany (2.27/5) and Spain (2.03/5). This situation is different for the group of young people with children, who gave the lowest relevance to having breakfast in family/ company (2.91/5 in Germany and 2.62/5 in Spain). In the UK, enjoyment and pleasure were ranked in the last position (2.30/5).

However, breakfast is attributed a very important role by most participants (57.8 %). All types of households associate it to “fuel” providing them with physical energy to cope with the day, except for the seniors, who strongly associate it with positivity and good mood to face the new day ahead.

### Places to have breakfast

The home stands out as the usual place for having breakfast (68.8 %) on weekdays, with an increase on weekends and public holidays (84.4 %). These are followed by those who have breakfast at work which has been brought in from home (20.2%). This habit is much more evident in young households, with (27.6 %) or those without children (36 %), particularly in the UK (21.6 %) and Germany (36.4 %).

### Origin of products consumed at breakfast

Breakfast products are usually bought in supermarkets. Note that homemade breakfast significantly increased in public holidays (20.2 %) vs. workdays (12.8 %). In addition to this, the use of products from other establishments increases significantly in public holidays (27.5 %) vs. weekdays (21.1%). These trends are reversed in the UK, where 21.2 % had homemade breakfast during the week, and 5.4% during weekends. Germany stands out for the use of organic products (30.3 %).

### Feelings linked to breakfast

There is a more positive emotional association (relaxation and enjoyment) on public holidays than on working days (loneliness and stress). In senior households, there is no such notable difference between the emotions evoked on workdays (6.25/10) vs. public holidays (7.90/10), as in other types of households: young without children (4.60 vs. 6.64/10), young with children (5.03 vs. 8.31/10), and mature with children (5.55 vs. 8.58/10).

### The ideal breakfast

Being able to have a more relaxed breakfast is valued as a priority, devoting more time to breakfast and broadening the variety of products consumed. This is one of the aspects participants give more relevance to in relation to breakfast (48.6%). In general, a greater availability of time does not imply a greater use of household appliances.

### Appliances used at breakfast

There is a generalized increase in the use of household appliances on public holidays. The toaster is the undisputed leader at breakfast (53.2 %), closely followed by the microwave/oven (49.5 %) and the coffee maker and the kettle (47.7 %), which are very popular in Germany and the UK. The use of pans / pots / griddle was ranked in second position during weekends (67.0 %), while during weekdays it fell to fifth position (31.2 %).

Four aspects are considered the most relevant in the use of appliances, regardless of whether they are used on weekdays or weekends: ease of cleaning, ease of use, contribution of a better taste and resistance/long duration ( Table 3).

**Table 3.** Results of the attributes identified for each smart cooking device from the online community. Question: For each of these attributes, in general, which device would you say is the most...(attribute)? Highlighted green values show attributes selected by > 50 % of the participants.

	Flatbread maker			3D food printer			*Sous-vide* cooking device		
	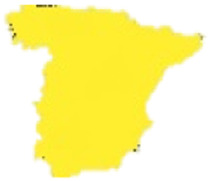	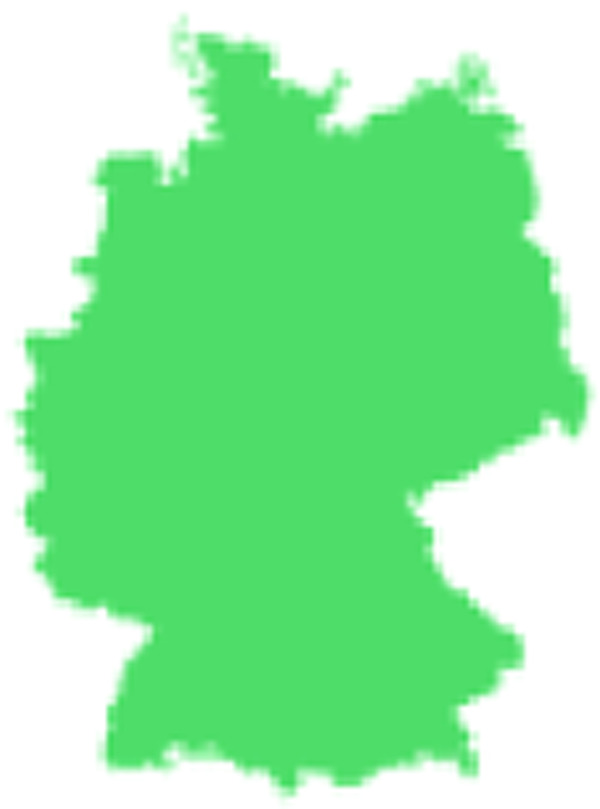	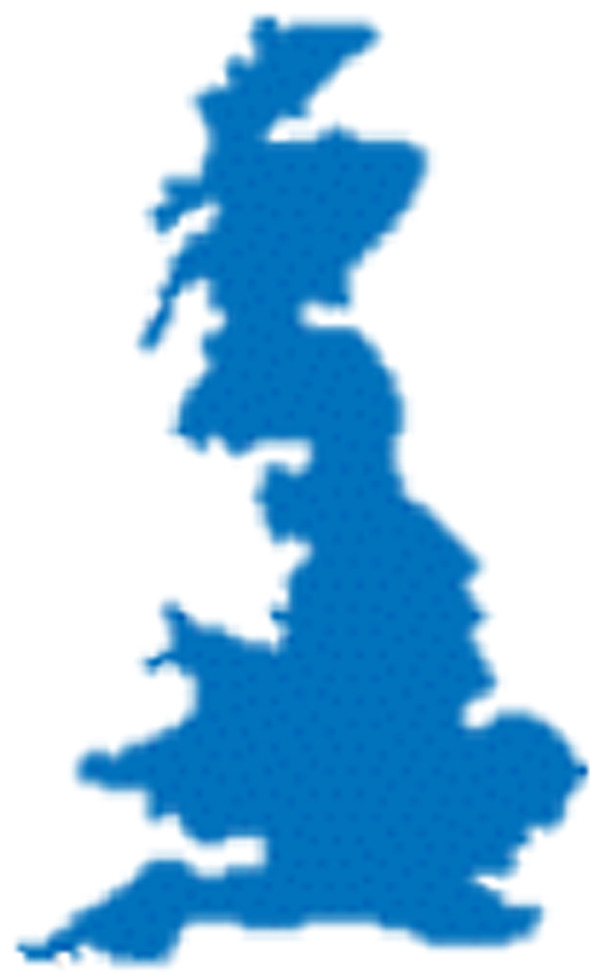	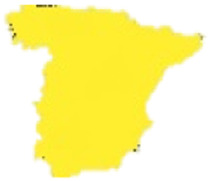	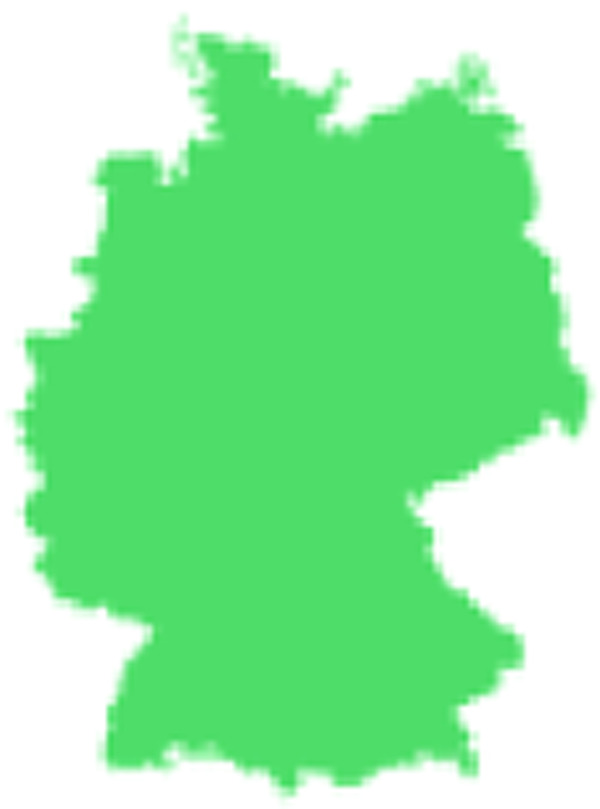	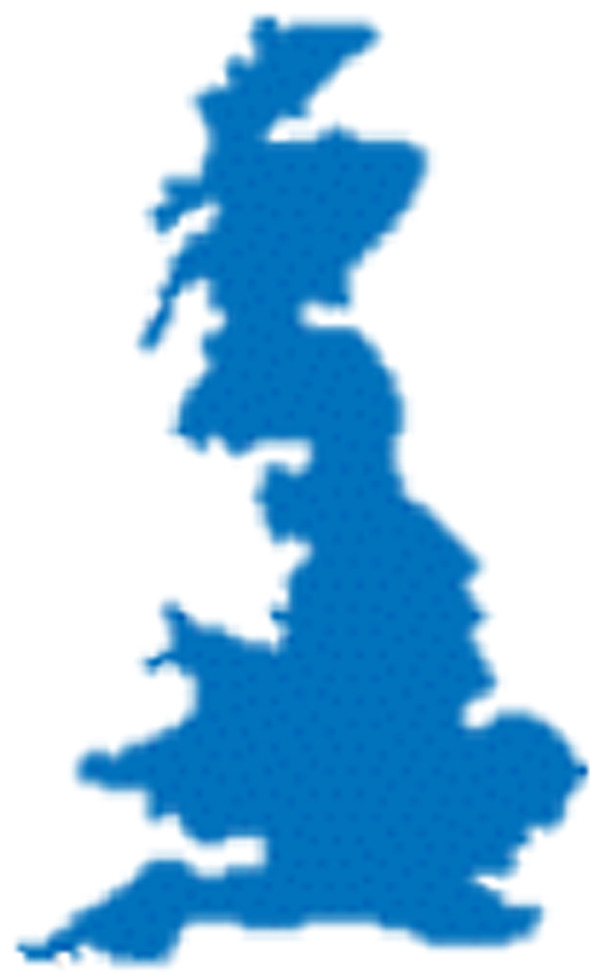	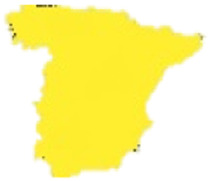	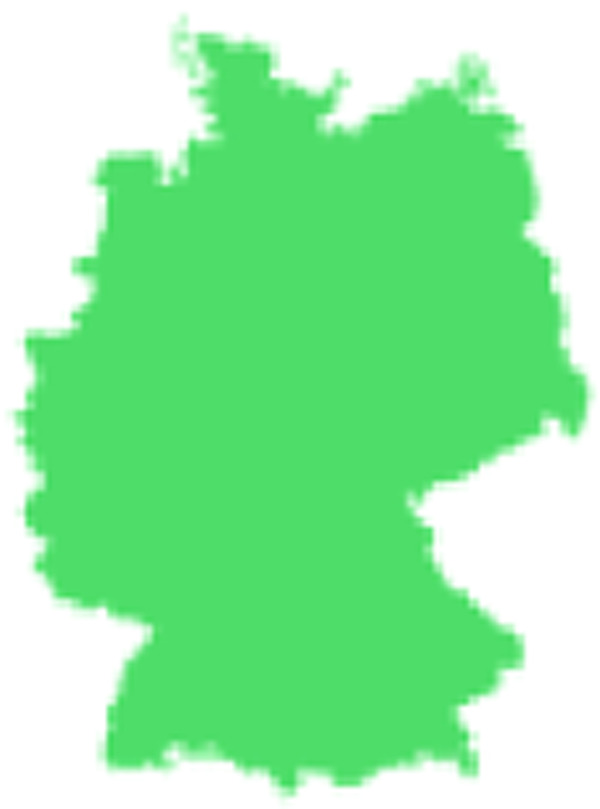	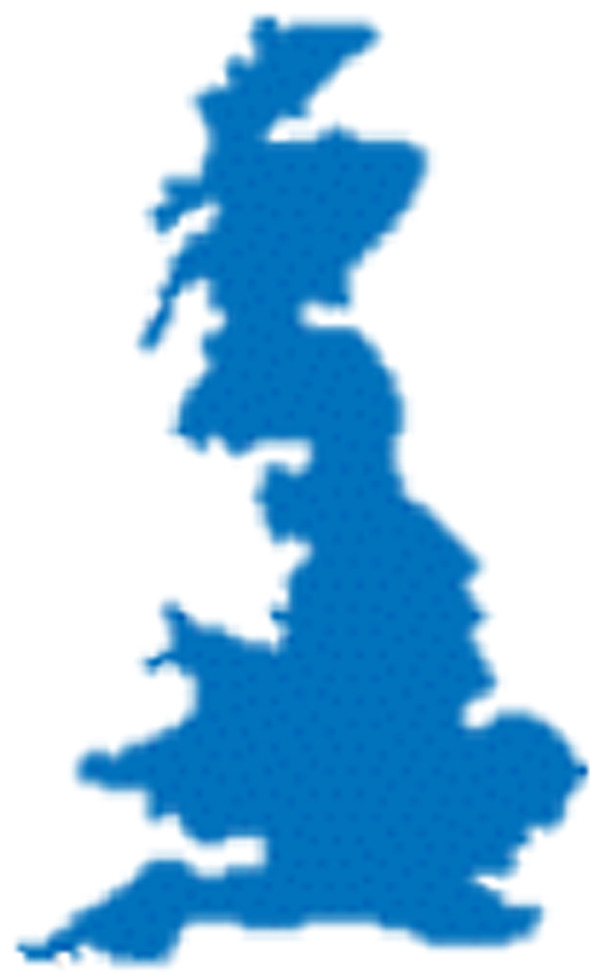
ATTRIBUTE	ES	DE	UK	ES	DE	UK	ES	DE	UK
Easy to clean	7.7%	8.8%	42.9%	10.3%	11.8%	2.9%	82.1%	79.4%	54.3%
Easy to use	51.3%	14.7%	88.6%	7.7%	5.9%	2.9%	41.0%	79.4%	8.6%
Functional / Practical	12.8%	17.6%	65.7%	28.2%	8.8%	17.1%	59.0%	73.5%	17.1%
Innovative	0.0%	20.6%	14.3%	82.1%	41.2%	82.9%	17.9%	38.2%	2.9%
Reliable	17.9%	23.5%	74.3%	25.6%	8.8%	8.6%	56.4%	67.6%	17.1%
Sustainable (ecologic)	15.4%	17.6%	31.4%	28.2%	14.7%	31.4%	56.4%	67.6%	37.1%
To prepare healthy food	2.6%	11.8%	37.1%	28.2%	20.6%	40.0%	69.2%	67.6%	22.9%
Useful	5.1%	11.8%	60.0%	28.2%	14.7%	17.1%	66.7%	73.5%	22.9%
Versatile	0.0%	8.8%	17.1%	35.9%	20.6%	54.3%	64.1%	70.6%	28.6%
With adequate size	0.0%	2.9%	42.9%	10.3%	8.8%	11.4%	89.7%	88.2%	45.7%

### Special foods

Around 50.5 % of households avoided products with sugar content. This behaviour stands out in mature and senior households (around 70 %). The second position has been taken up by lactose-free products in Spain (15.4 %), by gluten-free products in Germany (18.2 %) and by gluten-free products in the UK (16.2%).

### Healthy breakfast perception

In general, participants consider they have a healthy breakfast (7.24/10), although they think there is still room for improvement. In Spain, mature households with children were the most optimistic group (7.44/10). On the opposite side, young households with children were the most critical group in this context (6.96).

As a result of the online community, the type of household with the highest rating for each device per country, was selected as the target for each of the nine focus groups ( Table 4).

**Table 4.** Overall rating from 1 to 10 of the smart-cooking devices, based on what the consumers were able to know in the online community.

		Flatbread maker			3D food printer			*Sous-vide* cooking device		
TYPE OF HOUSEHOLD	AVERAGE RATING / type of household	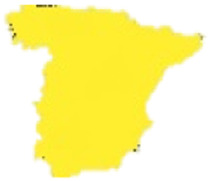	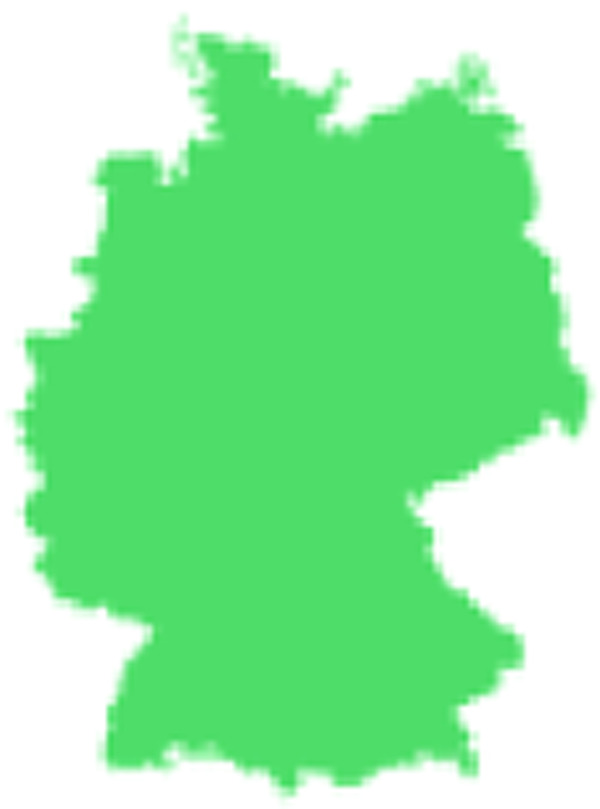	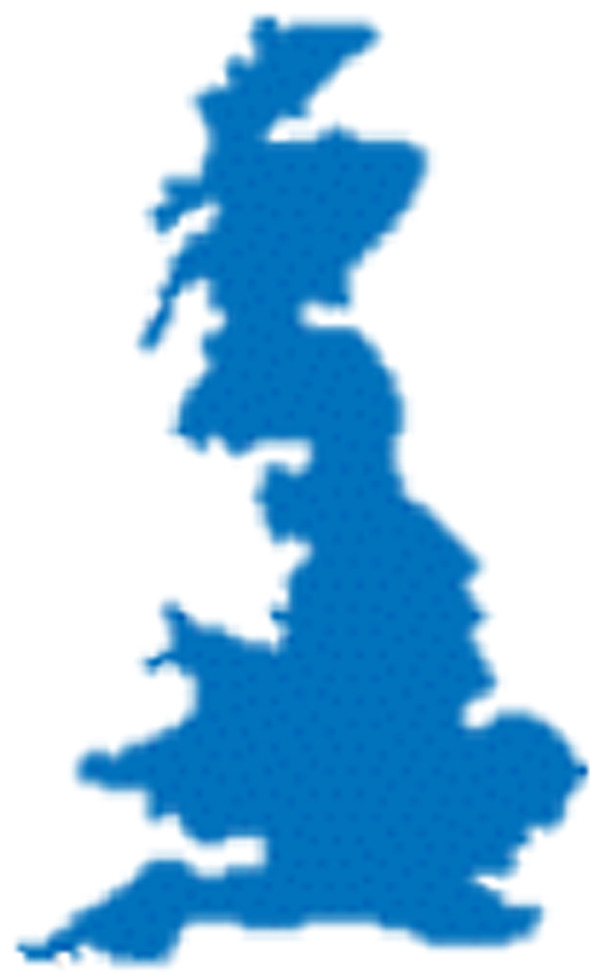	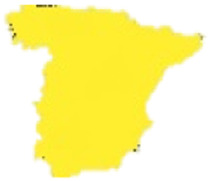	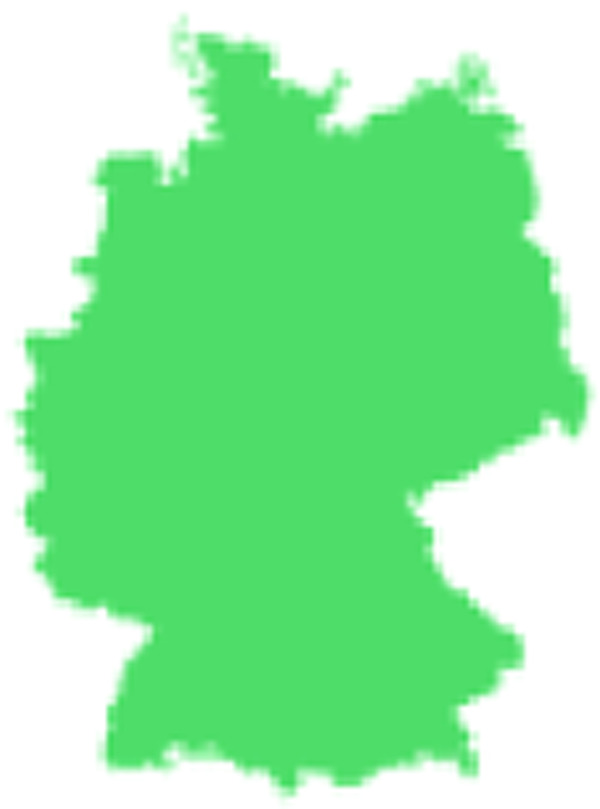	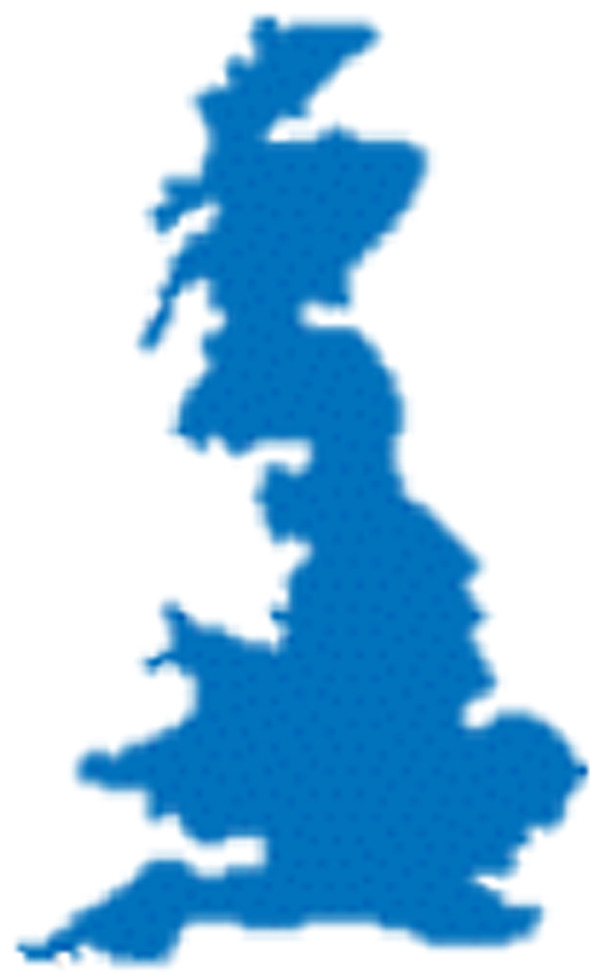	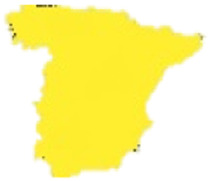	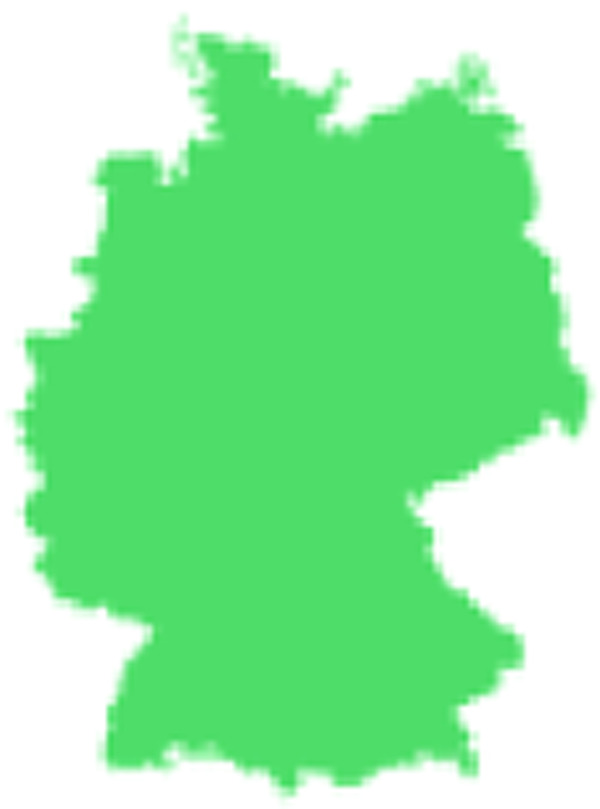	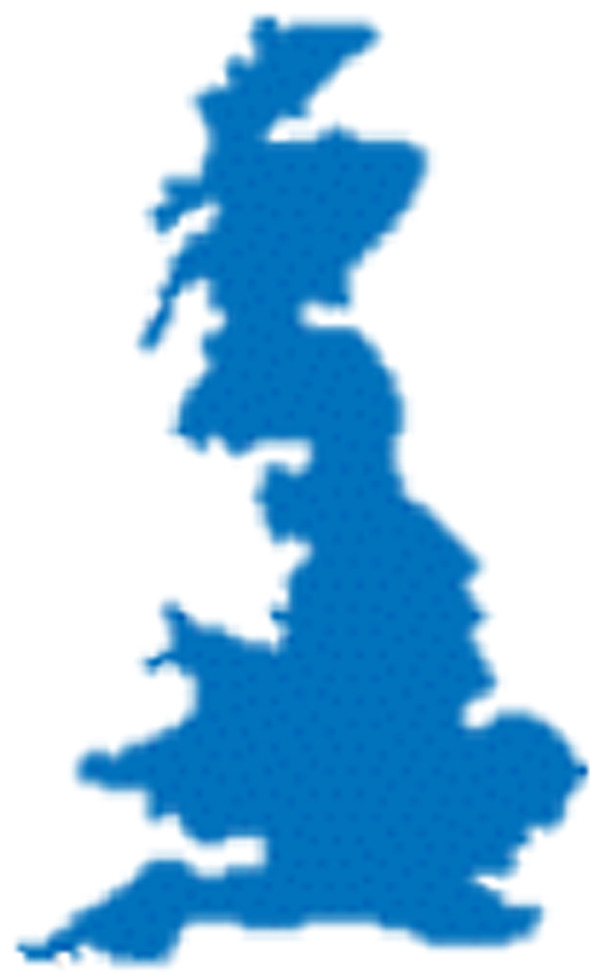
		ES	DE	UK	ES	DE	UK	ES	DE	UK
**Young without children** **(SINGLES)**	**7**	50.0%	42.9%	62.5%	40.0%	57.1%	25.0%	90.0%	42.9%	37.5%
		**6.8**	**6.7**	**7.0**	**7.7**	**6.0**	**6.00**	**8.3**	**7.3**	**7.0**
**Young with children** **(YOUNG FAMILIES)**	**6.9**	40.0%	20.0%	77.8%	50.0%	20.0%	33.3%	60.0%	90.0%	33.3%
		**7.0**	**4.5**	**7.3**	**8.6**	**6.0**	**7.3**	**8.8**	**7.7**	**5.3**
**Mature with children** **(CONSOLIDATED** **FAMILIES)**	**7.4**	60.0%	20.0%	55.6%	70.0%	30.0%	33.3%	80.0%	40.0%	11.1%
		**6.8**	**6.0**	**7.2**	**8.1**	**7.3**	**8.3**	**7.5**	**7.0**	**8.0**
**Senior**	**7.8**	44.4%	28.6%	55.6%	77.8%	14.3%	44.4%	77.8%	57.1%	44.4%
		**6.0**	**6.5**	**7.6**	**8.3**	**10.0**	**7.2**	**8.0**	**9.0**	**7.2**

Key - Percentage (%) represents positive responses to the question: *For each of the machines you have seen, choose one of the following options*: “Yes, I am interested”, “Yes, but not at this time”, “Yes, but it would depend on the final price that I do not know”, “No, in any case” (these respondents were removed for the analysis). - Overall rating of each device asked in the question: *What is your overall rating of [device] according to what you have been able to know in the online community?* - Highlighted cells indicate the type of households most interested (% positive responses or highest rating) in each device per country, and consequently, the targets selected for the nine focus groups, except for the sous-vide cooking device in UK.

For the 3D food printer, the target groups were as follows: *young without children* (Germany, 8 participants), *mature with children* (UK, 8 participants) and *senior* (Spain, 9 participants). For the *sous-vide* cooking device: *young without children* (Spain, 9 participants; UK, 9 participants) and *young with children* (Germany, 8 participants). Although senior consumers gave the highest rating and the highest percentage of positive responses, younger consumers were selected to ensure easy understanding of the instructions as this device was controlled from a mobile phone.

For the instant dough baking device, the target groups were as follows: *young without children* (Germany, 10 participants), *young with children* (UK, 8 participants) and *mature with children* (Spain, 9 participants).

During the focus groups, the appliances were tested by the participants who examined the products to be tested *in-situ*, in a real-life situation.

Thus, the product user behaviour, attitudes, and product problems and recommendations were evaluated. The focus group assessed how the appliances could be used in the preparation of meals. Consumers perceive such appliances as very useful and essential for preparing meals as they save time by avoiding some or all preparation steps. The most used appliances found in the kitchen were a toaster, coffee maker, mixer, sandwich maker, kettle, slow cooker, and juicer. These were considered the most versatile; the participants agreed that the microwave had lost its intended versatility. The less common kitchen devices are still used sporadically; the participants mentioned a cooking robot, raclette and fondue makers, waffle iron, rice cooker, crepe maker, blender, and bread maker. To make breakfast during the week, the participants preferred the appliances that stand out for their speed or simplicity, such as a toaster, coffee maker, microwave, and kettle. They also stated that their weekend breakfasts were different. During that part of the week, the slower appliances were used more often as the time spent at breakfast was longer than on weekdays. The most valuable attributes of the appliances mentioned by the users were utility, ease of use, ease of cleaning, speed, price, design, and size. The less important attributes were the energy consumption, additional cost, after-sales guarantee, durability, safety, and optimal result.

The results showed that, in general, the types of breakfast eaten on weekdays and during the weekends were different in all three countries. In Spain, breakfast during the week consists mainly of a beverage (coffee, tea, plain or flavoured milk), one bakery product (bread or cookies, in all their varieties), sometimes complemented by a yogurt, fresh fruit, or a fresh fruit juice (typically orange). During the weekends, more calorific products are often added (pancakes, fried or scrambled eggs, homemade bakery, etc.).

In Germany, there are also some differences between the weekdays and the weekends. During the week, a drink typically consumed for breakfast is coffee, milk, or tea (more frequent during the weekends). The beverage is accompanied by whole-wheat bread with either a savoury or sweet topping (sausage, cheese, butter, honey, jam, etc.). Less frequently, some vegetables or fruit are added (e.g., tomatoes, cucumbers). A more calorific breakfast is served during the weekend by increasing the portion size and adding some boiled eggs.

In the UK, the beverage does not vary on the weekdays (tea or coffee) and is complemented with toasted bread, oats (porridge), or breakfast cereals with milk, yogurt, or fresh fruit and orange juice. During the weekend, the typical English breakfast usually consists of fried eggs, bacon, beans, mushrooms, and toasted bread.

Several attributes were mentioned during the focus groups. The top attributes (with a greater presence in the speech) were utility, ease of use, ease of cleaning, speed, price, design, size. The attributes with less presence were neat, safety, optimal result, energy consumption, additional cost, after-sales guarantee, durability.

**Figure 2.**
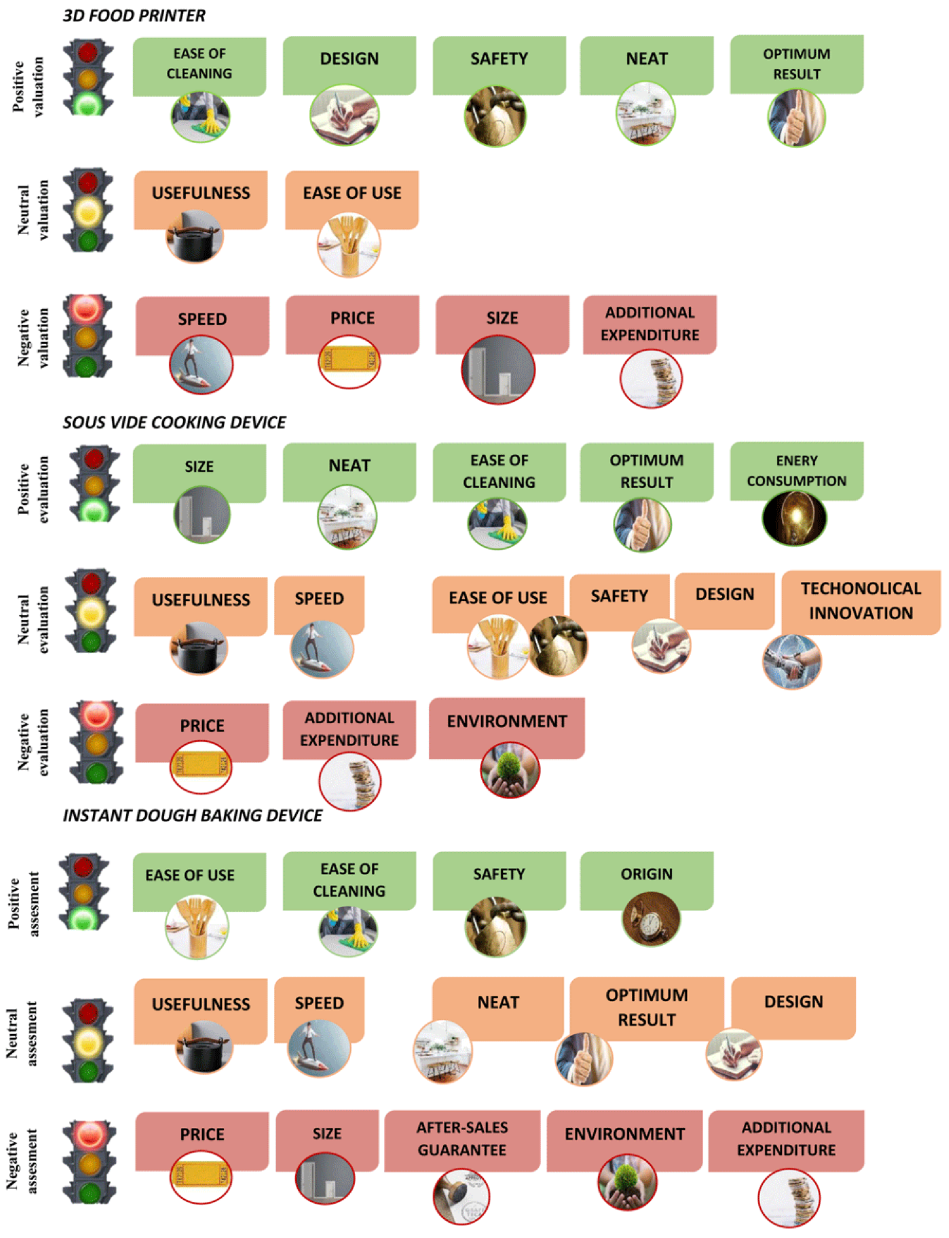
Attributes perceived for each smart cooking device in terms of traffic light colour: green as positive validation, orange as neutral validation and red as negative validation.

The attributes perceived for each smart cooking device are shown in Figure 2 in terms of traffic light colours: green as positive validation, orange as neutral validation and red as negative validation. Results show that the 3D food printer had five positive attributes (ease of cleaning, design, safety, neat and optimum results), two neutral (usefulness, ease of use) and four red (speed, price, size, additional expenditure). The *sous-vide* cooking device had five positive attributes (size, neat, ease of cleaning, optimum result, energy consumption), six neutral (usefulness, speed, ease of use, safety, design, technological innovation) and three red (price, additional expenditure, environment). Finally, the instant dough baking device had four positive attributes (ease of use, ease of cleaning, safety, origin), five neutral (usefulness, speed, neat, optimum result, design) and five which were considered negative (price, sales, after-sales guarantee, environment, additional expenditure).

Results of the quantitative study remain confidential to the start-ups as they show purchase intention and influence of initial selling price for each device. However, it was interesting to see the differences which were found regarding the ideal place for each cooking device ( Table 5). In the case of the *sous-vide* cooking device and the instant dough baking device, it was obvious that the ideal place was at home (58.4 % and 42.4 %, respectively). However, for the 3D food printer, the ideal place was in a restaurant (39.3 %).

**Table 5.** Results for the ideal place for each smart cooking device per country of the online survey. Highlighted cells indicate the highest % of responses to the question: *Where do you think each of these appliances you have just seen, fits best? Only one answer.*

	3D food printer				Sous-vide cooking device				Flatbread maker			
	TOTAL	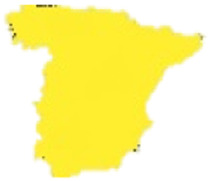	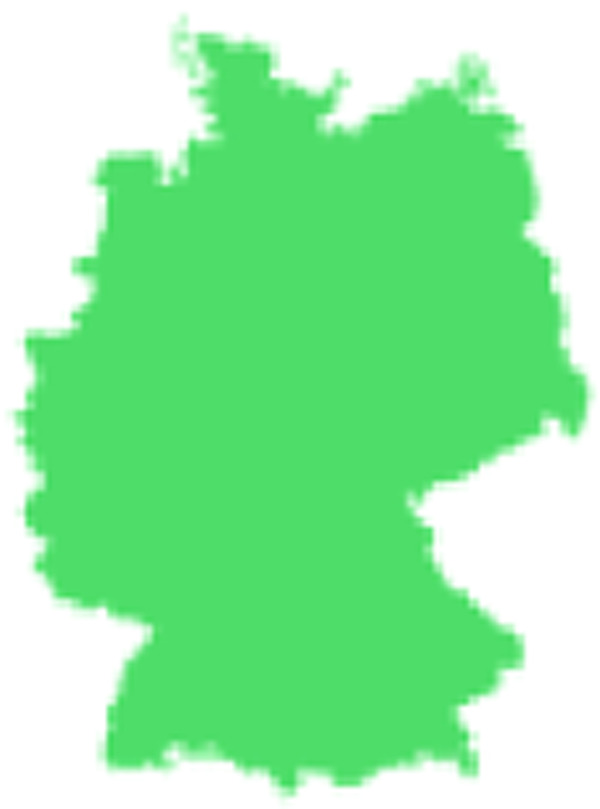	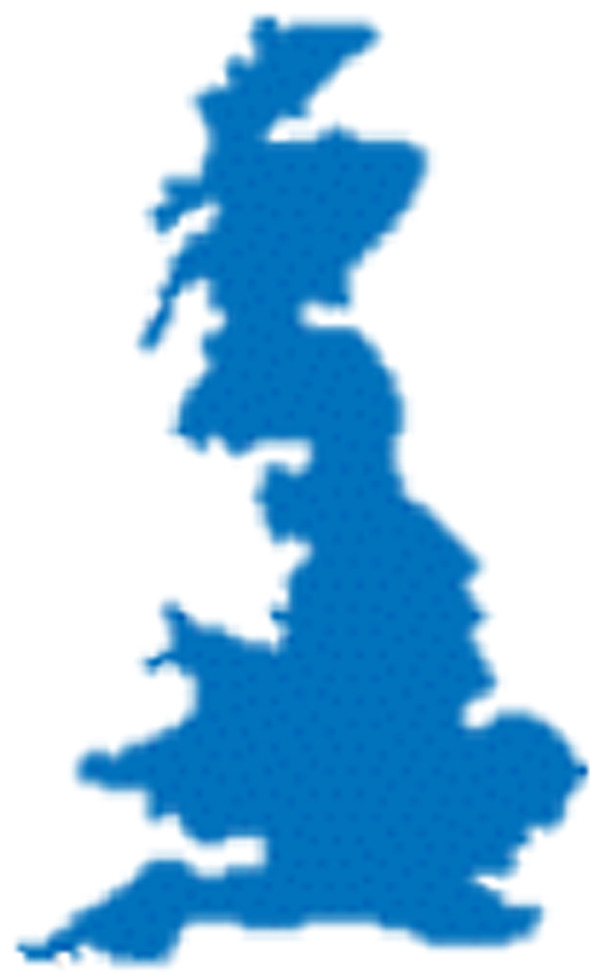	TOTAL	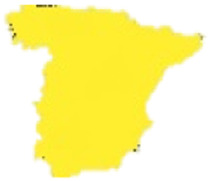	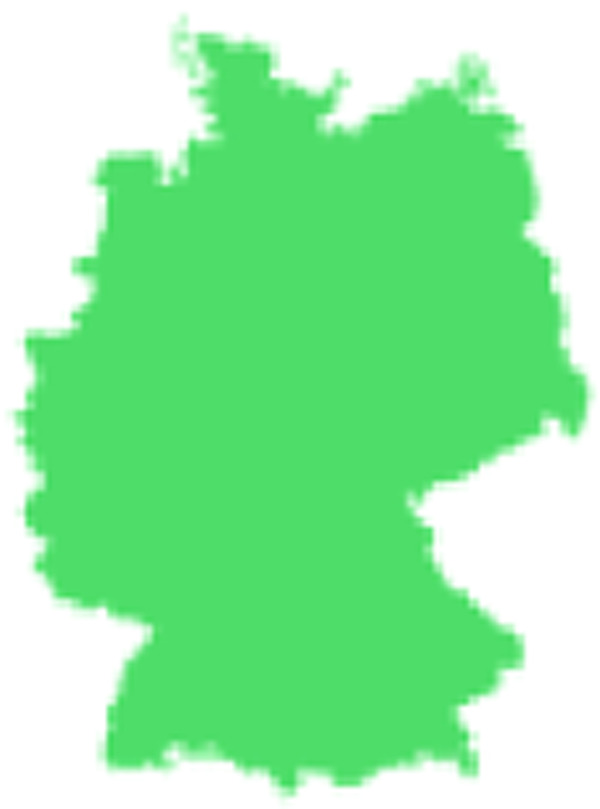	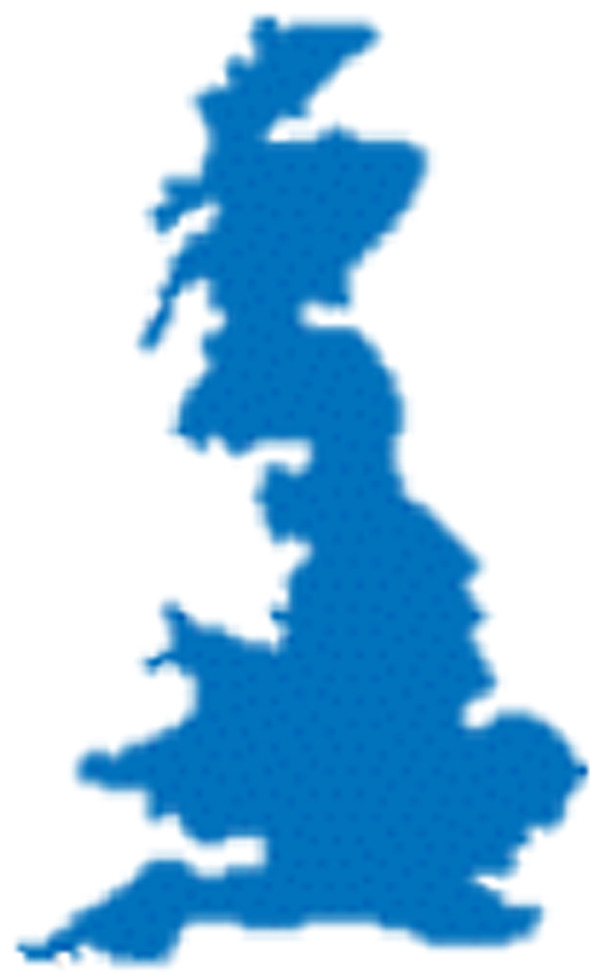	TOTAL	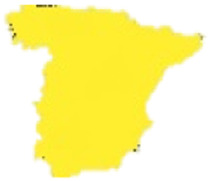	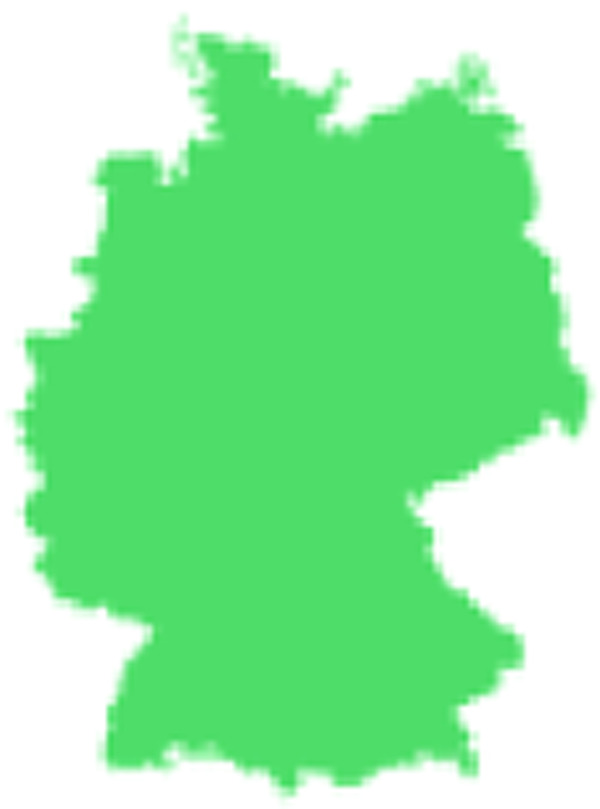	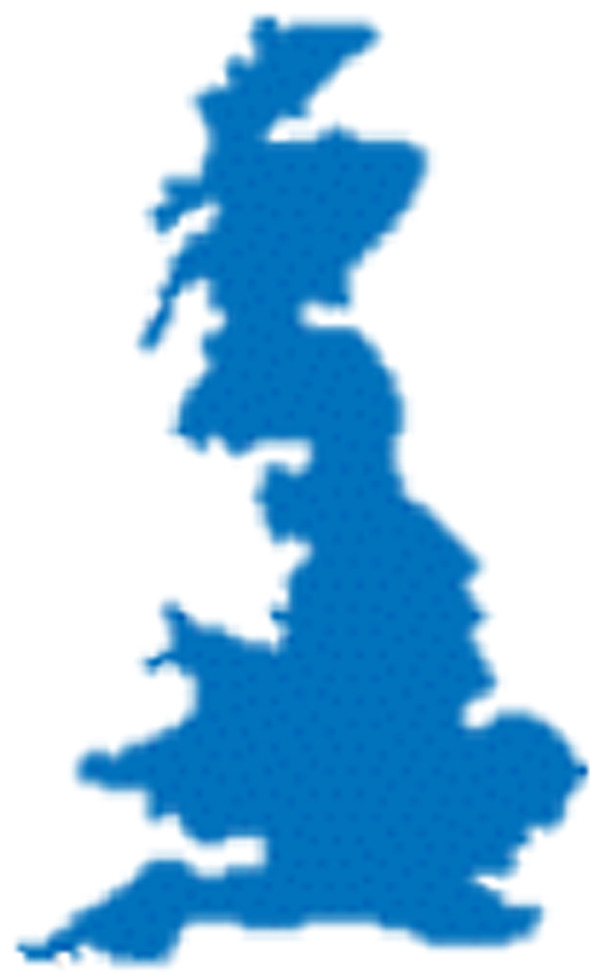
… at home	23.7	20.6	20.7	29.7	58.4	60.6	54.6	60.0	42.4	40.0	36.2	50.9
… in a workplace	12.8	12.6	10.9	14.9	9.2	8.6	13.2	5.7	11.6	14.3	13.8	6.9
… in a restaurant	39.3	40.6	40.2	37.1	12.8	9.7	9.8	18.9	13.9	11.4	9.8	20.6
… in a bar	6.9	4.6	12.6	3.4	4.6	3.4	6.3	4.0	12.0	12.6	17.2	6.3
… in a hospital	3.1	4.6	1.7	2.9	4.6	7.4	4.0	2.3	0.8	1.1	1.1	--
… in a nursing home	2.5	5.1	0.6	1.7	4.4	3.4	6.3	3.4	1.0	0.6	1.1	1.1
… in a school	1.5	2.9	1.1	0.6	1.7	2.3	0.6	2.3	2.3	1.7	2.9	2.3
… in a bakery	5.3	4.0	4.6	7.4	0.8	1.7	0.0	0.6	5.7	8.0	2.3	6.9
… in a buffet	5.0	5.1	7.5	2.3	3.6	2.9	5.2	2.9	10.3	10.3	15.5	5.1
*Bases*	*524*	*175*	*174*	*175*	*524*	*175*	*174*	*175*	*524*	*175*	*174*	*175*

Results from the interview with the experts also identified restaurants as the ideal place for the 3D food printer, as opposed to the instant dough baking device and the *sous-vide* device, which were visualized more in work offices such as in common areas for coffee and breakfast.

With regards to the elderly care sector, which suffers from serious shortages of skilled staff a growing demand for fast and straightforward methods for preparing specialized foods was acknowledged by the interviewed experts. Moreover, the general upward trend in allergies and intolerances translates into an increased need for specialty foods. The three smart cooking devices were perfectly placed to fulfil such needs. The elderly residents of care homes have shown willingness to actively participate in their diet choices ( Bell *et al*., 2020), which would place an increased variety of food options at their disposal, especially for the individuals with chewing or swallowing problems.

While some elderly care facilities specialize in difficulties in swallowing and chewing, they only comprise a small fraction of such facilities. During this study, all care homes approached were very aware of the challenge, and either have elderly residents with these issues or have had them in the past.

Using the SmartBreakfast approach could help people with chewing or swallowing problems to access a wider variety of foods by providing the products with a proper edible texture. Thus, a normal eating experience can be restored, as the prepared food resembles traditional dishes in its appearance, smell, and taste. This may encourage the residents to eat larger quantities of food more often and increase their perceived quality of life. The improved food intake results in the desired weight gain, preventing the potentially dangerous malnutrition and helping in recovery from any current disorders. Moreover, the residents with chewing and swallowing problems can eat the same food as “normal” residents, which could improve their mood and prevent social exclusion. This last aspect was highlighted as being important by experts in the elderly care sector. The potential for mood improvement and the enjoyment component (also known as "fun factor”) associated to preparing your own breakfast with a smart device, coupled with increased variety and range of food options, could also positively impact the staff in these facilities.

### Co-design of complementary food formulations for a common value offer

Results from the quantitative study were used to quantify the relevance of breakfast, the most valued nutrients, the places to have breakfast, and the appliances frequently used to prepare it. For the question “ *How important are, in your opinion, the different meals you eat every day?*”, 89.7% of Spanish participants said breakfast, 97.7 % lunch and 71.4% dinner. Among German consumers, 85.1 % considered breakfast important, 84.5 % lunch and 73.6 % dinner. In UK, breakfast relevance was rated a bit lower, 80.0 %, lunch 80.6 % and dinner 92.6 %.

As the purpose of this study was to obtain insights for the moment of breakfast consumption, the results obtained from the questionnaires will focus only on breakfast.

For the questions, “ *Which nutrients would you consider the most important in your breakfast?*”, fibre was top rated in Spain (49.7 %) and UK (39.4 %) whereas in Germany vitamins were chosen as the most important nutrient for breakfast (44.8 %).

For the question “ *Where do you usually have your breakfast*?”, the majority of participants for the three countries responded “at home” during working days (75.6 %) and holidays/weekends (83.4 %). It was important to notice that in Germany, during working days, 23.6 % of participants had breakfast at work and 62.6 % at home. This percentage was much higher during weekends (91.4 %). In Spain, during holidays/weekends 14.9 % of participants claimed to have breakfast in a bar/coffee shop/ bakery, and 78.3 % at home.

Finally, to the question “ *of the following appliances, which ones do you usually use for breakfast?”*, the toaster and the coffee makers were the most popular for the three countries (66.4 % and 55.7 %, respectively, during working days; and 70.6 % and 55.5 %, respectively during holidays/weekends). In Spain, also the juicer (62.3 %) and the microwave oven (39.4 %) were chosen after the toaster (77.7 %) during working days, with slightly no difference with respect to weekends/holidays. In UK, the kettle (65.7 %) was as popular as the toaster (65.1 %), and the coffee maker was less relevant (29.1 %). No difference was found among working days and holidays/weekends.

Considering the results of the qualitative and quantitative studies discussed above, AZTI developed a prototype using the *sous-vide* cooking device, the 3D food printer and the food ingredients from the industrial ingredient supplier. In particular, the mean scores for the use of the smart cooking devices per type of household ( Table 4) from the online community already showed that the type of household with the highest average scores for the three devices was the senior household (7.8/10). The average for single households was 7.0, 6.9 for young families and 7.4 for consolidated families. Therefore, the senior household was selected as the target group for product development and commercialisation synergies among the three start-ups.

**Figure 3.**
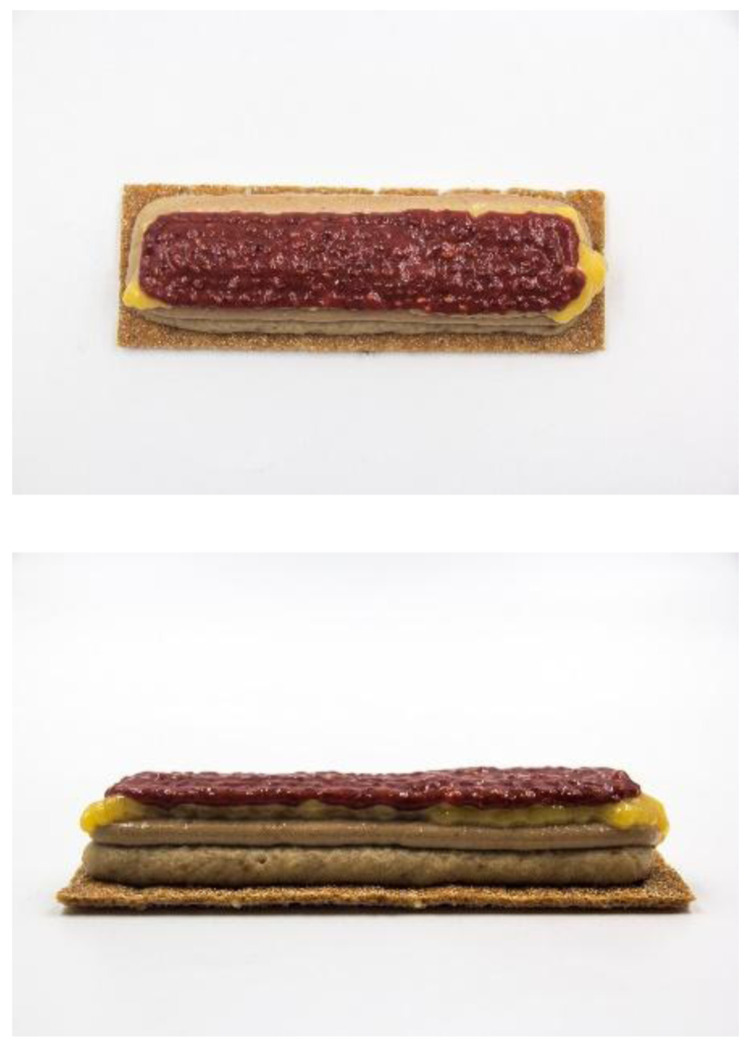
3D printed breakfast bar designed for senior consumers using the smart cooking devices.

This prototype was a cereal bar concept produced by using the three devices ( Figure 3): first the dough was automatically baked from a capsule by using the instant dough baking device, then four edible inks were *sous-vide* cooked at a range of temperatures from 70 to 85°C, over a range of time from 10 to 40 min. Finally, they were loaded in five different capsules of the 3D food printer. Two capsules of 40 mm nozzle diameter, two capsules of 15 mm nozzle diameter and one capsule of 8 mm nozzle diameter were used. A basic rectangular design was loaded in the software of the 3D food printer. For each type of ink parameters such as print speed, line thickness and distance between layers were adjusted.

The ingredients used of each edible ink were:

1. Oat ink: milk (50 %), raisins (9 %), banana (21 %), oats (20 %)
2. Nut ink: milk (32 %), nut paste (57 %), egg yolk (11 %)
3. Egg ink: pasteurized egg (80 %), milk powder (20 %)
4. Strawberry ink: concentrated strawberry puree (90 %), chia seeds (10 %)

Nutritional analysis of the breakfast bar was carried out, and nutritional claims could be made ( Table 6): *contains naturally occurring sugars*, *source of protein*, *low in saturated fat*, *reduced caloric value*, *high in unsaturated fat*, *high in polyunsaturated fat*. 

**Table 6.** Nutritional profile of the 3D printed breakfast bar.

Nutrient composition	g/100g
Moisture content	54.3
Carbohydrates	26.4
Sugar	7.6
Protein (Nx6.25)	7.0
Insoluble dietary fibre	2.2
Fat	8.7
Saturated fat	1.6
Sodium	0.1
Salt (from sodium)	0.3
Ashes	1.4
Caloric value (kcal)	216.5
Caloric value (Kjul)	908.2
FATTY ACIDS PROFILE	
Saturated	18.42
Monounsaturated	21.49
Polyunsaturated	60.10
Trans fatty acids	0.18
Omega 3	10.94
Omega 6	49.16
C4:0 butyric acid	0.54
C6:0 caproic acid	0.31
C8:0 caprilic acid	0.13
C10:0 (capric acid)	0.31
C11:0	<0.10
C12:0 (lauric acid)	0.39
C13:0	<0.10
C14:0 miristic acid	1.36
C14:1n-5t Mirestelaidate acid	<0.10
C14:1n5c myristoleic acid	0.15
C15 (pentadecanoic acid)	0.17
C15: 1n5c	<0.10
C16:0 palmitic acid	11.46
C16:1n7c palmitoleic acid	0.45
C17: 0 margaric acid	<0.10
C17:1n7c margaroleico acid	<0.10
C18:0 stearic acid	3.64
C18:1n12t petroselaid acid	<0.10
C18:1n9t elaidic acid	<0.10
C18:1n7t transcendent acid	0.18
C18:1n-12c petrosenyl acid	<0.10
C18:1n9c oleic acid	19.23
C18:1n7c vacanic cis acid	1.02
C18:2n6t linoelaidic acid	<0.10
C18:2n6 (9c12t)	<0.10
C18:2n6 (9t12c)	<0.10
C18:2n6c linoleic acid	49.16
C20:0 arachidic acid	0.11
C18:3n6c linolenic range (GLA) acid	<0.10
C18:3n3c alpha linolenic aq. (ALA)	10.94
C20:1n9c gadoleic acid	0.46
C21:0	<0.10
C20:2n6c	<0.10
C 22:0 (Behenic acid)	<0.10
C20:3n3c	<0.10
C20:3n6c DHLA	<0.10
C22:1n9c euricic acid	<0.10
C20:4n6c arachidonic acid	<0.10
C23:0	<0.10
C22:2n6c	<0.10
C20:5n3c EPA	<0.10
C24:0 lignoceric acid	<0.10
C24:1n9c Nervous acid	<0.10
C22:5n3c clupadononico acid	<0.10
C22:6n3c cervonic acid (DHA)	<0.10

*Highlighted cells indicate nutrient contents that allow nutritional claims

**Figure 4.**
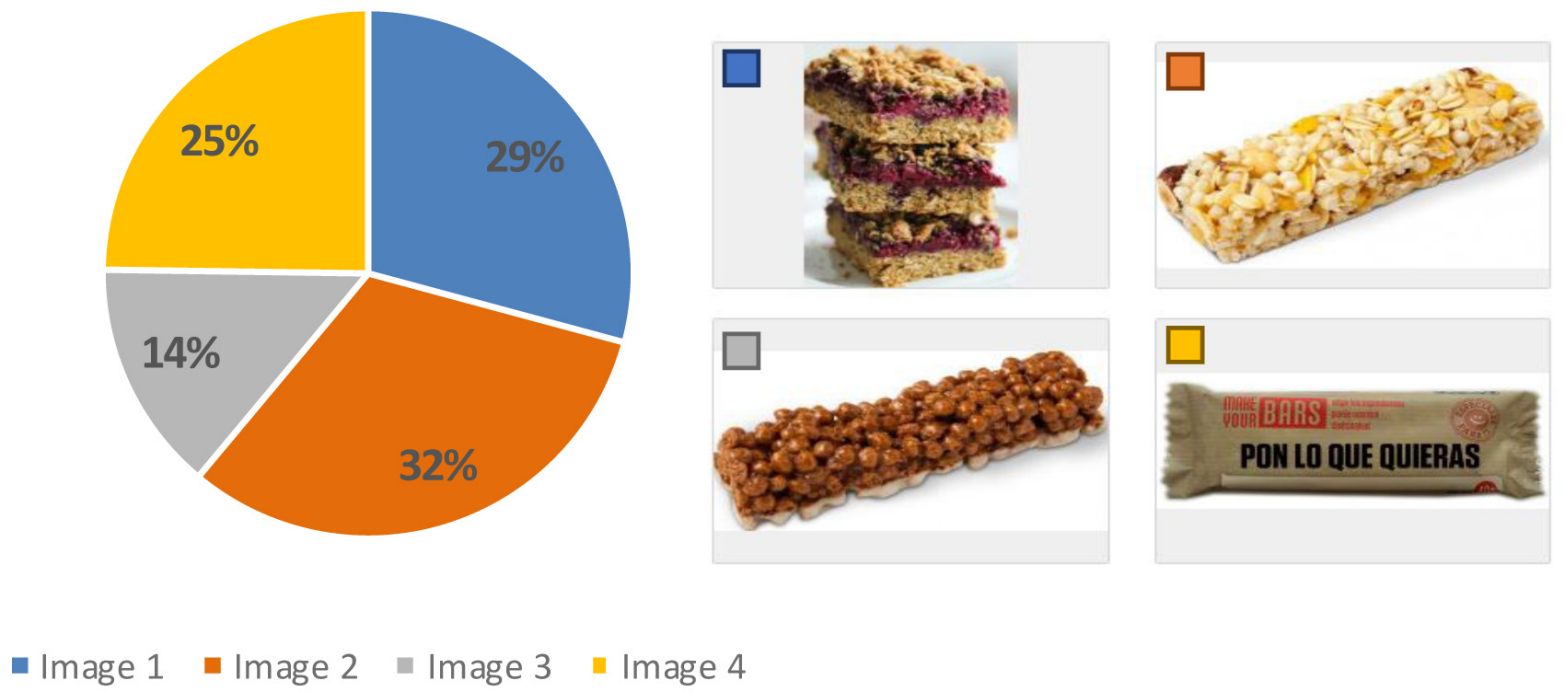
Percentage of answers to the question “What do you think you’re going to try?” (n = 80).

**Figure 5.**
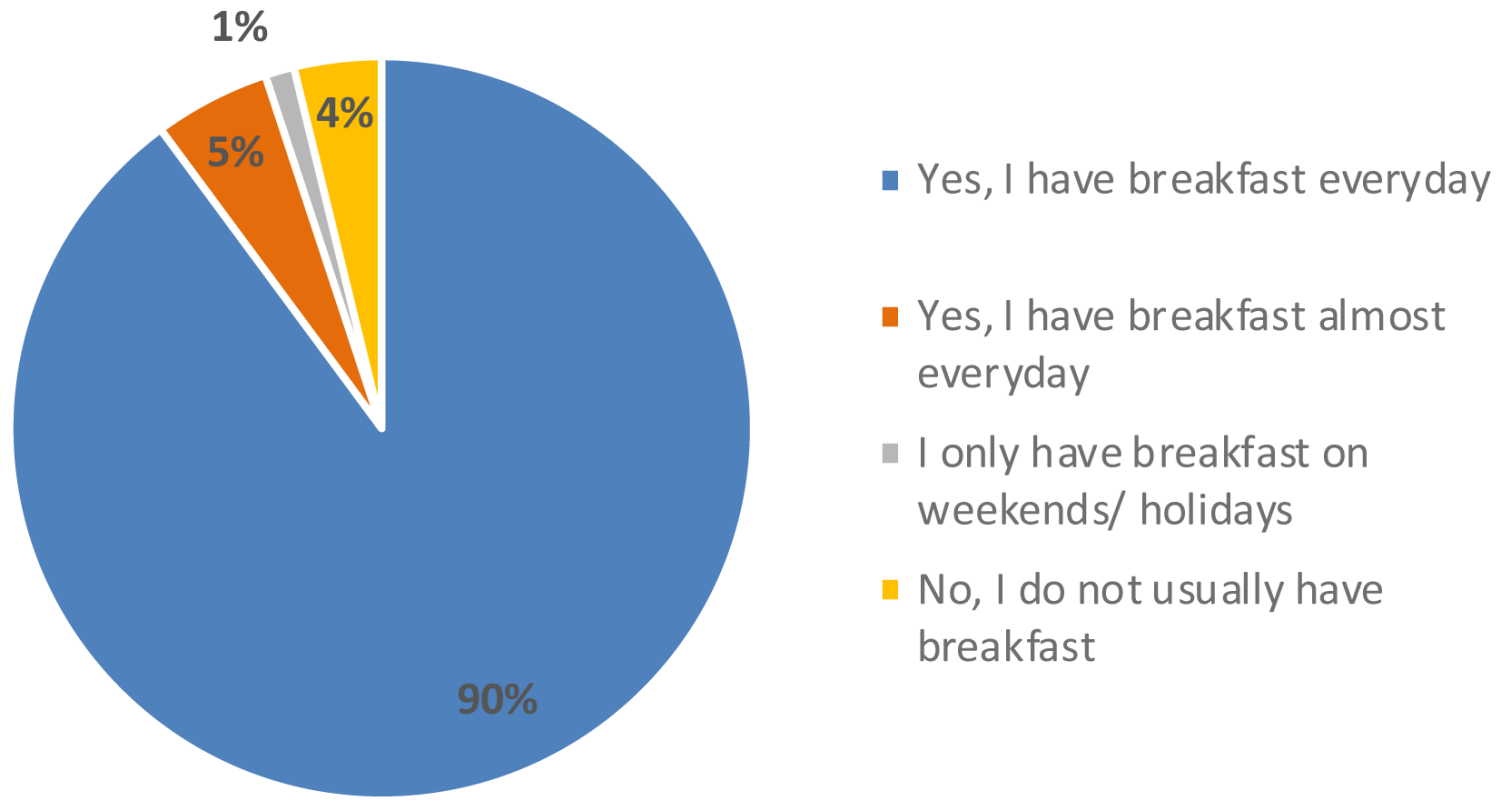
Percentage of answers to the question “Do you usually have breakfast?” (n = 80).

Spain was the country with the highest average rates (from Table 4) for the use of all the devices (7.6) compared to Germany (7.0) and UK (7.1), therefore, the sensory analysis and acceptability test was carried out in Spain (AZTI facilities). Results from this test showed that before showing the product, more than 50 % of the participants imagined a cereal bar with fruit, 32 % thought of a more industrial appearance and 29 % considered a more home-made appearance ( Figure 4). Regarding their eating habits ( Figure 5), 90 % of the participants ate breakfast every day and 4 % did not usually have breakfast. Among the foods most consumed at breakfast there were bread with jam, oil or sausage (15 %), cookies (12 %), fruit (12 %), cereal flakes (11 %) and dairy products (11 %). Among the least consumed were cereal bars (1 %) and industrial bakery (1 %). Results indicated that only knowing the concept and appearance, the acceptability was 6.0 ± 2 points, which means, the expectation of the senior consumer was “I will like it slightly”. When testing the product, the acceptability decreased significantly to 5.0 ± 2 (t = 3.071, p-value = 0.003 **) with texture being the variable that most influenced the acceptability of the product (4.8 ± 2), followed by aspect (5.2 ± 2), aroma (5.7 ± 2) and taste (5.0 ± 2). The consumer considered that the concept did not fit the product she/he had tasted and that the texture was too soft, pasty and dense, which was unpleasant in the mouth. This might be since the texture of the breakfast bar was designed for senior residents with potential swallowing problems. The senior consumers recruited were not residents of an elderly care facility. Therefore, the advantages of a texturized product which is easy to swallow were not perceived as a positive attribute.

**Figure 6.**
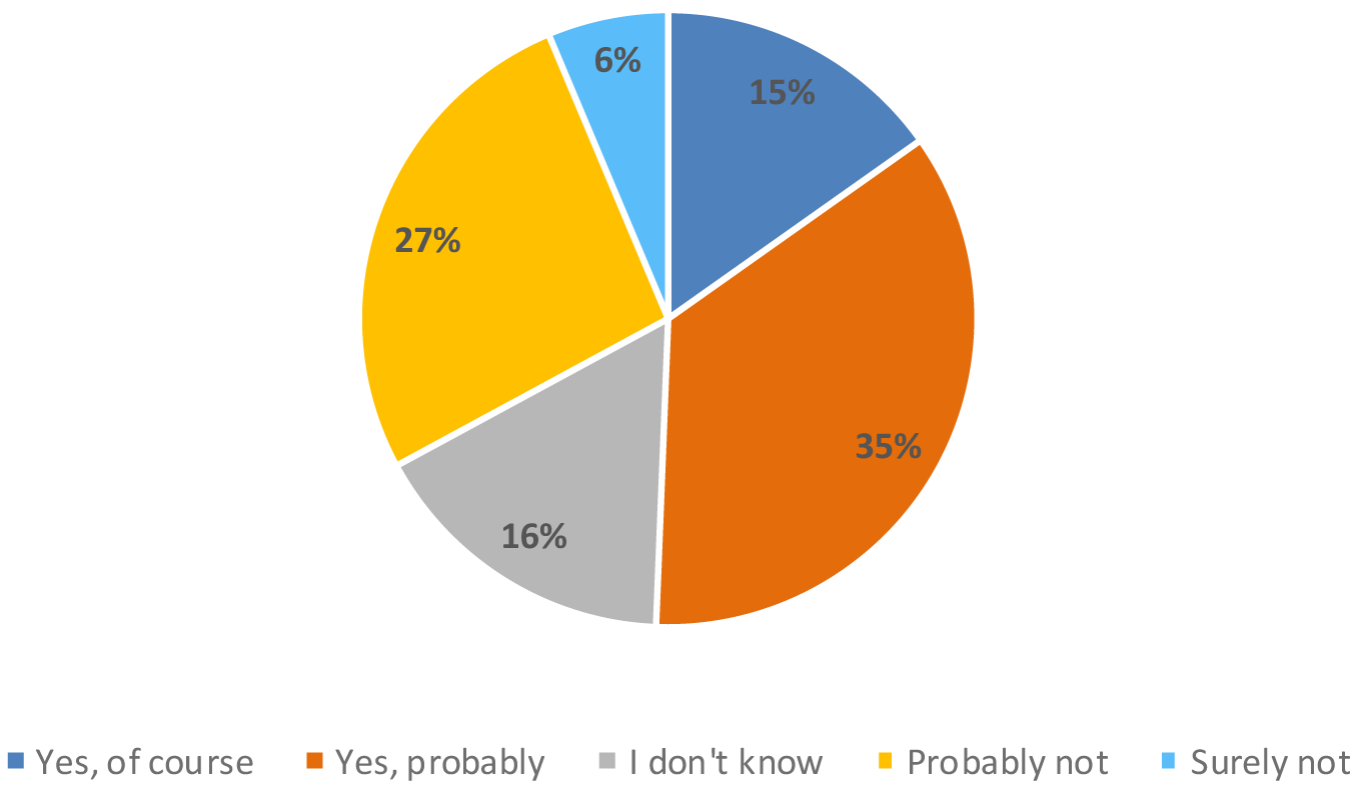
Percentage of answers to the question “Do you think that this new appliance could be useful in your home?” (n = 80).

However, consumers considered 3D printing technology innovative (18%), industrial (17%), modern (13%) and accurate (12%). Around 50% of the participants in the study believed that a 3D printer could be useful in their homes, compared to 33% who thought it would not be useful ( Figure 6).

**Figure 7.**
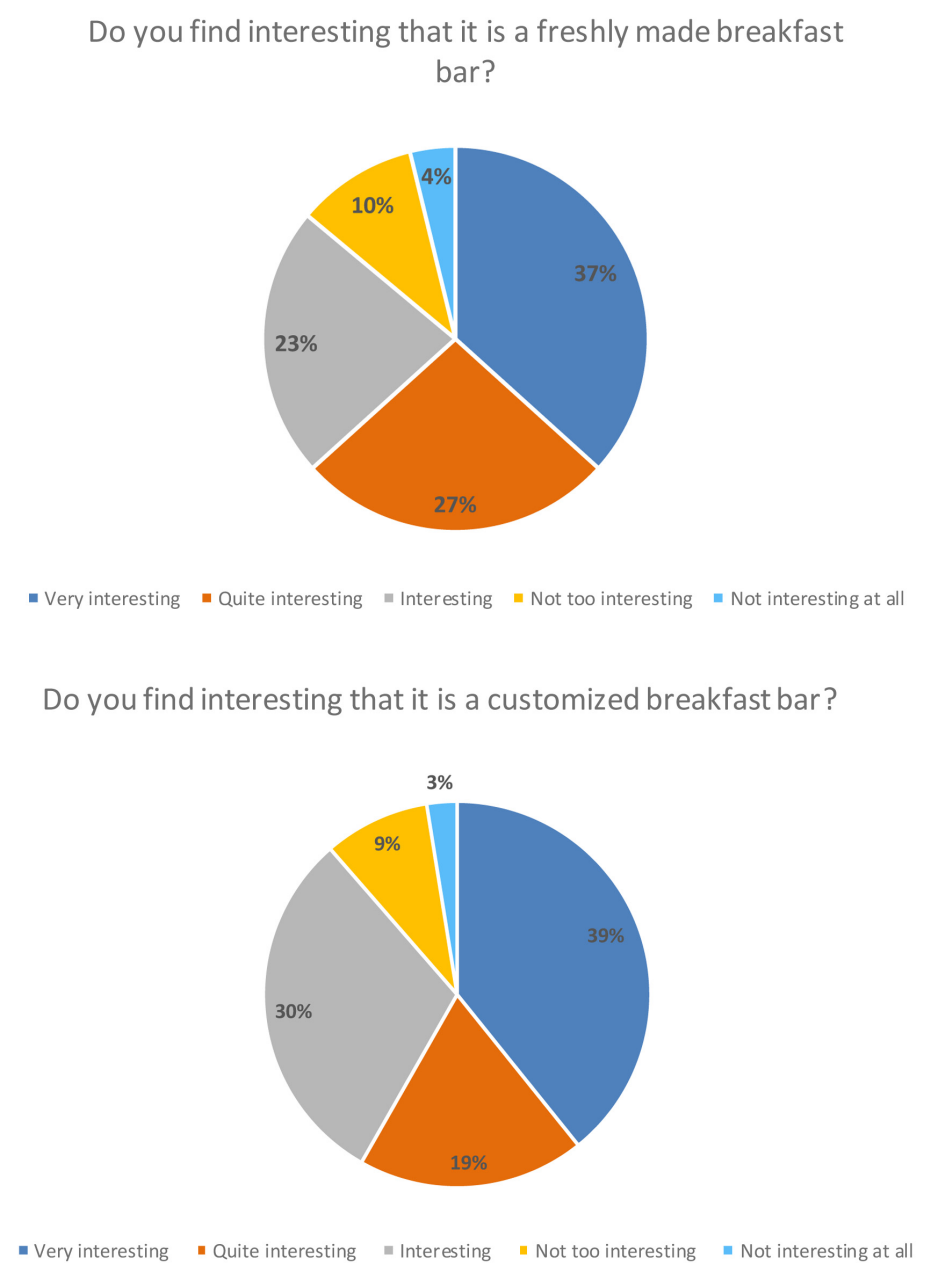
Percentage of answers to the question “Do you find interesting that it is a freshly made breakfast bar?” and “Do you find interesting that it is a customized breakfast bar?” (n = 80).

Finally, more than 80 % of the consumers participating in the study considered it interesting that it was a freshly made breakfast bar that was personalized, meaning that they could choose the ingredients and the design of the product themselves ( Figure 7).

**Figure 8.**
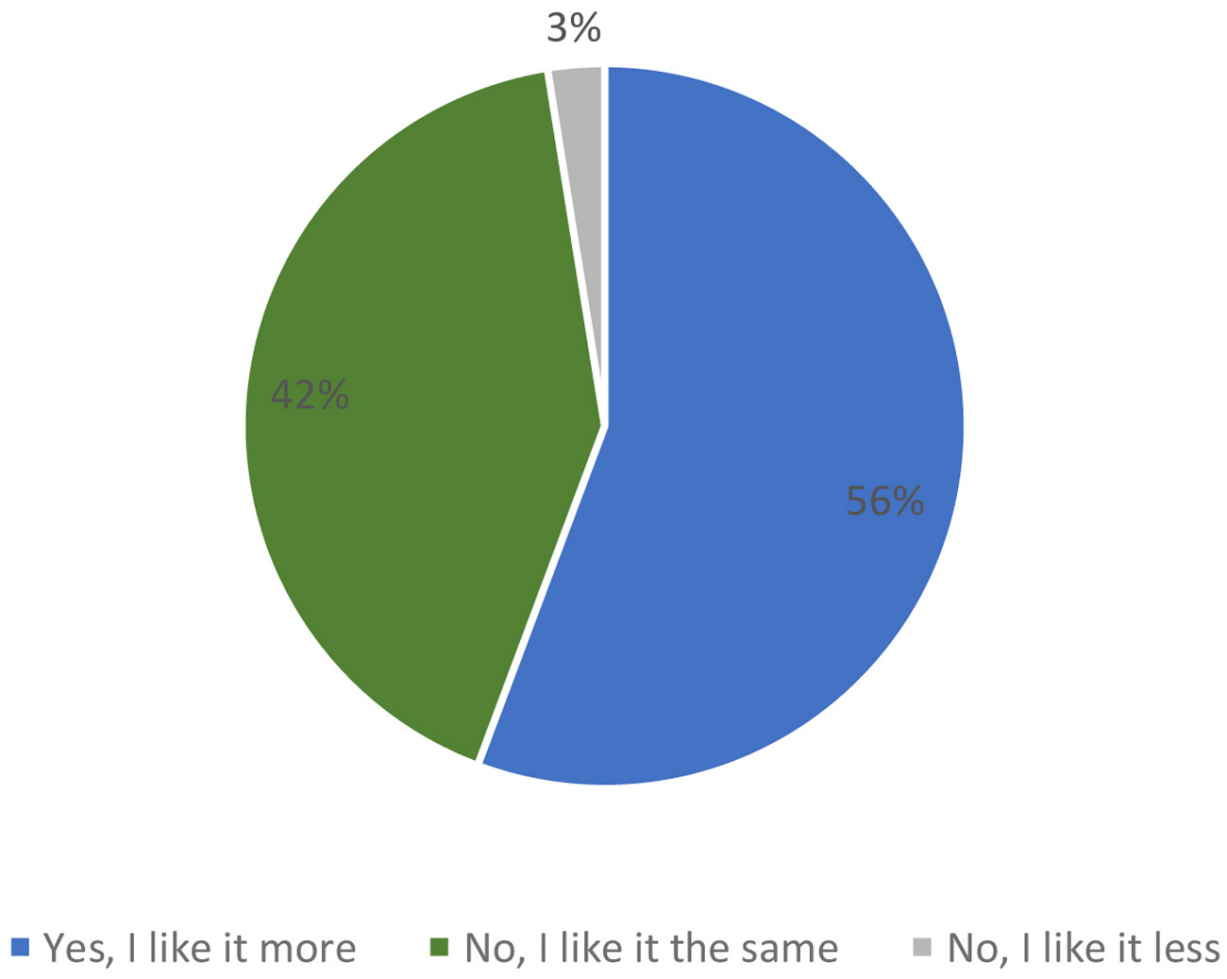
Percentage of answers to the question “If we say this breakfast bar is source of protein, low in saturated fat and with reduced caloric value, do you like the product more?” (n = 80).

When consumers were informed that it was a bar with the following nutritional claims: “source of protein”, “low in saturated fat” and with “reduced caloric value”, 56 % of participants said they liked the product more versus 42 %, which did not change its acceptability. Only 3 % of consumers said that they liked the product less when they knew the information about the ingredients ( Figure 8).

## Discussion

Overall, findings from these research show that technology can help consumers make informed, personalized nutrition choices using direct engagement, giving them access to new product concepts and informative packaging. Likewise, the home appliance industry presents emerging opportunities to improve consumer nutrition, increase supply-chain efficiency and transparency, and boost productivity and profitability ( Guo & Zheng, 2019; Newswire, 2018). The use of appliances is driven mainly by convenience; therefore, increasing or upgrading appliance capabilities based on consumer feedback will improve the interaction and engagement of consumers.

For the success of start-up companies, often developing new technologies, understanding consumer perception and stakeholders’ opinions are vitally important. Such knowledge allow them to efficiently use their scarce resources ( Laage-Hellman *et al*., 2018). Business accelerator programs can help in the formulation of initial uses cases and identify the target customer segments. The customers are the best source of information on the desirability of the product that a start-up should produce ( Laage-Hellman *et al*., 2018).

## Conclusion

The findings from this study provided both theoretical and practical insights into the perception of the three smart cooking devices per type of household and per country. A combination of technologies was used to develop the new breakfast concept for the target group and country with the most positive perception. The set of method used in this study show how to share resources for gathering information on product attributes and consumer experience, and for validating the new concept with the target group identified via consumer market research.

Cooperation between new food-tech enterprises can create innovative collaboration schemes, between start-ups, research institutions, industrial partners and consumers. This promotes further development and increases the use of new technologies in the food sector while meeting the expectations of society in an increasingly efficient way. Moreover, such collaboration offers opportunities to consider novel solutions for achieving important goals in improving human health, sustainable production and consumption, and tackling climate change.

## Data availability statement

The data that support the findings of this study are available (as project deliverables) upon providing sound, sensible and fair reasons for request to the corresponding author, with prior consent of the project partners. Due to the nature of this research (consumer behaviour on specific devices), the business companies involved in this study did not agree for the data to be shared publicly as it contains information about prototypes not commercially available when the study was carried out. Improved versions have been developed since then, so additional supporting data of the current study is not available.

### Extended data

ZENODO: Talens, Clara, Santa Cruz, Elena, & Rios, Yolanda. (2021). SmartBreakfast project: New Food Products for Innovative Home Appliances

10.5281/zenodo.5748270 ( Talens *et al.,* 2021)

Data file 1: Moderator guide for the SmartBreakfast online community

Data file 2: Video 1_SmartBreakfast project – EIT Food partners, objectives and result

Data file 3: Video 2_3D printer Breakfast Bar

Data file 4: Video 3 Senior consumer test of a 3D breakfast bar

Data are available under the terms of Creative Commons Attribution 4.0 International (CC0 4.0 Attribution 4.0 International).

## Footnotes

^1^ Information on the three start-ups can be found at the EIT Food – SmartBreakfast website.
