# Supplementary material for: Leveraging innovative technologies for designing a healthy and personalized breakfast: consumer perception of three smart cooking devices in the EU
Source: Open Res Eur. 2022 May 3;1:151. Originally published 2021 Dec 14. [Version 3] doi: 10.12688/openreseurope.14234.3 (PMC10521106; doi:10.12688/openreseurope.14234.3)
Supplement: Peer review history

# Reviewer report: Reviewer response for version 3

**DOI:** 10.21956/openreseurope.15950.r29138

**Published:** 2022-05-09

**Authors:** Jessica Aschemann-Witzel

**Affiliations:** 1 MAPP - Centre for Research on Customer Relations in the Food Sector, Aarhus University, Aarhus, Denmark

The authors have adequately addressed my concerns and I can now approve this article.

If applicable, is the statistical analysis and its interpretation appropriate?

Yes

Is the study design appropriate and is the work technically sound?

Yes

Is the work clearly and accurately presented and does it engage with the current literature?

Partly

Are the conclusions drawn adequately supported by the results?

Yes

Are sufficient details of methods and analysis provided to allow replication by others?

Partly

Are all the source data and materials underlying the results available?

Partly

Reviewer Expertise:

NA

I confirm that I have read this submission and believe that I have an appropriate level of expertise to confirm that it is of an acceptable scientific standard.

# Reviewer report: Reviewer response for version 2

**DOI:** 10.21956/openreseurope.15679.r28572

**Published:** 2022-03-01

**Authors:** Jessica Aschemann-Witzel

**Affiliations:** 1 MAPP - Centre for Research on Customer Relations in the Food Sector, Aarhus University, Aarhus, Denmark

Dear authors,

 Thank you very much for your revisions of the article. I agree with these except for the first point. Let me explain this in the following.

 I do not think that the problem that I have raised is solved by a mere change of word from ecosystem to collaborative innovation.

 What I see as a problem is that the article is still shifting in focus from one section to the next. It starts out with a research question that is not looked at, uses methods and results fitting to a different research question, and then concludes on the first research question, even though this has not been researched. As I am asked to assess the validity of the article content, I have to conclude that I do not think the article has a valid content in the way it is presented now. That does not mean I think it is not valuable and publishable, on the contrary, but I think the good results presented are embedded in the wrong research question and conclusion.

 The results are about consumer behaviour insights about the technologies and the products that came out of the different technologies used. The methods used are consumer behaviour methods to explore consumer perception and reaction. But the question that is asked in the title and in the research question mentioned in the abstract and in the introduction is about exploring the capabilities of value chain actors in co-creating innovation. The title also says that the method is a case study. The conclusion says that the collaborative innovation has shown that the sharing of resources can encourage entrepreneurship and help start-ups define their target group. But this is not what had been explored and shown.

 There is not really a proof in the data shown, that supports the conclusion that this way of collaborating is leading to better results than others (if the question is quantitatively approached), or of which type and direction the capabilities are that are leveraged, or what is the mechanism observed for leveraging the capabilities as emerging from the data or the type of synergies created (if the question is qualitatively approached). How do you prove your conclusion that the collaboration provides new synergies if what you show is not about the collaboration of the actors and their synergies but about the consumer research results? I acknowledge that the consumers are an active actor as well, but the data is not about the synergy and collaboration because it focuses on the consumer perceptions of the technologies and products – not about all actors perceptions of the collaboration process.

 I therefore still suggest to change the title, the research question, and conclusions in all places where mentioned to something that fits the research question asked in the plain language summary (‘This work analysed what consumers perceived …’), instead of the ones in the introduction (‘scrutinizes customer involvement in product development using an process-based single case study approach’, ‘investigate the capability of an innovation ecosystem to co-create …’).

 The only place where I see a method with results that could fit to the current focus is the eight expert interviews mentioned on page 7, because this might contain questions on the capability in the collaboration and entrepreneurship potential. But I do not see results from these interviews that reflect the focus question, and it is also only a small share of the data.

 Apologies for making things more complicated by not being satisfied. A different researcher might come to a different conclusion on this issue that I raise and might not see the problem of mismatch that I see. However, from my point of view this does not work and cannot do otherwise.

If applicable, is the statistical analysis and its interpretation appropriate?

Yes

Is the study design appropriate and is the work technically sound?

Yes

Is the work clearly and accurately presented and does it engage with the current literature?

Partly

Are the conclusions drawn adequately supported by the results?

Yes

Are sufficient details of methods and analysis provided to allow replication by others?

Partly

Are all the source data and materials underlying the results available?

Partly

Reviewer Expertise:

NA

I confirm that I have read this submission and believe that I have an appropriate level of expertise to confirm that it is of an acceptable scientific standard, however I have significant reservations, as outlined above.

# Response

**Published:** 2022-04-25

**Authors:** Clara Talens

**Affiliations:** AZTI, Food Research, Basque Research and Technology Alliance (BRTA), Spain

Dear Jessica,

 We would like to acknowledge once more your valuable comments, and we highly appreciate having the opportunity to submit a new version according to the latest comments. We have gone through the manuscript and recognised that there is indeed a mismatch between the title, the research question raised and the conclusion, perhaps due to a lack of specificity and a too ambitious approach.

 We have now adapted the title, abstract, main objective, discussion and conclusion to a more realistic approach, bearing in mind the content of the study. We have stuck to the content of the results, which are, (i) the in-depth study of the consumer perception of 3 smart home cooking devices, and (ii) the tasting session. We have also re-written the abstract, objective and conclusions in the different sections to be consistent throughout the manuscript.

 We hope with this change, the article’s scope is better described, and the results better match the research question raised. Best regards

# Reviewer report: Reviewer response for version 1

**DOI:** 10.21956/openreseurope.15352.r28226

**Published:** 2022-01-24

**Authors:** Jessica Aschemann-Witzel

**Affiliations:** 1 MAPP - Centre for Research on Customer Relations in the Food Sector, Aarhus University, Aarhus, Denmark

The research paper ‘Leveraging capabilities for the creation of a smart, healthy and personalized breakfast: a case study of innovation ecosystems in the EU’ describes a multi-method consumer study in three countries to assess the acceptability of three new technologies for use in breakfast.

 The paper is based on quite a lot of consumer research data and of different types, thus it has a rich database. It is a very interesting approach to study the different technologies in these consumer research method steps as well as across countries.

 However, I have a few points of critique. I am unsure about the requirements for the publication outlet, so I assess the paper as I would in other scientific journals of the food field.

1. The most critical issue is that there is a problem in the match between the data used and presented as results, and the question asked. The research question seems to be about how an innovation eco-system can be efficiently designed and how it can be created to deliver good insights, but the data presented is about what consumers think about these technologies for their breakfast. If the paper would be about the first, the data and method would need to be e.g. about what are innovation eco-systems, comparing such eco-systems, analysing how the eco-system worked, interviews with the companies about the collaboration, etc.
  My suggestion would be to change it to the latter, that is, to focus on the consumer research. That would mean to change the title, and to change the abstract and introduction in every place where the purpose of the paper is described, and also focus the conclusions on the results of the answers to the question about consumer acceptance of these technologies.
2. There is no discussion section. This is usually a crucial element of a scientific research paper, to set the results in the context of previous research. I suggest to scrutinize the results for sentences that in fact go in the direction of discussing the findings (there are some comments on what it means and how to interpret it), move these to a new section called discussion, and then write a paragraph at minimum of the length of the current conclusion with at least a few references to previous research on new food technologies/breakfast /consumer acceptance, discussing what the findings mean in comparison to theory and literature.
3. A rich and multi-method data set often has the problem that the reader gets a bit lost about all the different methods and steps. I suggest that a figure with the methods could help, and/or a subdivision of the methods with a heading for each method step.

A number of smaller points are as follows:

- Rather do not write cases when you mean the consumer sample, write participants (else it can be confused with a case study).
- There are a few definitions one could add. For example, what is meant by ‘smart home’.
- The following statement (the second sentence of the abstract, also in the introduction) is unclear: " **In the long run the smart home appliance industry will reflect the social, technological, and demographic forces around food without losing the authenticity of food traditionally prepared in the kitchen**." Why is that, and who says this will happen, based on which argument? I would suggest explaining this more in the introduction.
- Figures 3 ff should indicate the sample size.

If applicable, is the statistical analysis and its interpretation appropriate?

Yes

Is the study design appropriate and is the work technically sound?

Yes

Is the work clearly and accurately presented and does it engage with the current literature?

Partly

Are the conclusions drawn adequately supported by the results?

Yes

Are sufficient details of methods and analysis provided to allow replication by others?

Partly

Are all the source data and materials underlying the results available?

Partly

Reviewer Expertise:

Consumer research in the food area.

I confirm that I have read this submission and believe that I have an appropriate level of expertise to confirm that it is of an acceptable scientific standard, however I have significant reservations, as outlined above.

# Response

**Published:** 2022-02-05

**Authors:** Clara Talens

**Affiliations:** AZTI, Food Research, Basque Research and Technology Alliance (BRTA), Spain

The authors would like to acknowledge the valuable comments of reviewer Jessica Aschemann-Witzel. We have created a new version incorporating the suggested changes. Please, find below responses to each comment in *italics*.

1. The most critical issue is that there is a problem in the match between the data used and presented as results, and the question asked. The research question seems to be about how an innovation eco-system can be efficiently designed and how it can be created to deliver good insights, but the data presented is about what consumers think about these technologies for their breakfast. If the paper would be about the first, the data and method would need to be e.g. about what are innovation eco-systems, comparing such eco-systems, analysing how the eco-system worked, interviews with the companies about the collaboration, etc.
  My suggestion would be to change it to the latter, that is, to focus on the consumer research. That would mean to change the title, and to change the abstract and introduction in every place where the purpose of the paper is described, and also focus the conclusions on the results of the answers to the question about consumer acceptance of these technologies. 

*Response: We have replaced the word ecosystem by “collaborative innovation” so the focus is given to the advantages of collaborative innovation to understand consumer acceptance of new technologies.   *

1. There is no discussion section. This is usually a crucial element of a scientific research paper, to set the results in the context of previous research. I suggest to scrutinize the results for sentences that in fact go in the direction of discussing the findings (there are some comments on what it means and how to interpret it), move these to a new section called discussion, and then write a paragraph at minimum of the length of the current conclusion with at least a few references to previous research on new food technologies/breakfast /consumer acceptance, discussing what the findings mean in comparison to theory and literature.

*Response: A discussions section has been created relating the findings to the theoretical background on the home appliance industry and strategic collaborative relationships.* 

1. A rich and multi-method data set often has the problem that the reader gets a bit lost about all the different methods and steps. I suggest that a figure with the methods could help, and/or a subdivision of the methods with a heading for each method step.

*Response: A figure has been added at the beginning of the article to explain the methodology followed during research.* A number of smaller points are as follows:

- Rather do not write cases when you mean the consumer sample, write participants (else it can be confused with a case study). 

*Response: The word 'cases' has been replaced by 'participants'.*

- There are a few definitions one could add. For example, what is meant by ‘smart home’.

*Response: 'Smart home' has been removed from the text, authors believe that 'smart devices' is easily understood.*

- The following statement (the second sentence of the abstract, also in the introduction) is unclear: "In the long run the smart home appliance industry will reflect the social, technological, and demographic forces around food without losing the authenticity of food traditionally prepared in the kitchen." Why is that, and who says this will happen, based on which argument? I would suggest explaining this more in the introduction.

*Response: The sentence has been rephrased.* 

- Figures 3 ff should indicate the sample size.

*Response: Sample size has been included in each figure.*

# Reviewer report: Reviewer response for version 1

**DOI:** 10.21956/openreseurope.15352.r28180

**Published:** 2022-01-05

**Authors:** Gyula Kasza, Tekla Izsó

**Affiliations:** 1 National Food Chain Safety Office, Budapest, Hungary

The paper reports on an interesting and very complex study in an actual topic with cross-cultural aspects implementing a multidisciplinary approach. Besides being a valuable international comparative research, one of the major strengths is the cooperation of the entrepreneurs from the private sector and academics, also the large sample for consumer testing.

 Despite the fact that the experimental design and the interpretation of the used methods are adequate, the wide focus poses some challenges for the authors and the readers, as well. A flow chart or infographics indicating the sequence and interdependencies of the different research stages would provide significant support for better comprehension.

 Some minor additions and changes in the description of the methodology may improve the understanding of the study such as:

- The breakfast types of the selected countries should be edited in a table to indicate the differences and similarities among them.
- The description of the sampling goals should be explained (representativity aspects also) in the Methodology section.
- A description of the pilot testings would improve the scientific quality.
- The "collaboration model" should be emphasized better in the introduction and research design parts.

The results are presented accurately, however, in some points clarification would be needed. Answering and checking the following questions in the text of the report and completing this section would be advantageous (but not essential):

-  In the "Special foods" section: Were "wheat-free" and "gluten-free" different claims in the study? Or does this refer to an open-ended question? Or is it just a translation bias?
- An explanation of the "multi-channel tool" would be great.
- The paper contains only a few references to other studies. I recommend identifying similar RDI projects from the food sector and comparing their findings (and the methodological approach) to the actual study.

It is understandable that the research data is available in part due to the business interests of the participating companies. Nevertheless, the findings are supported by the outcome of the research.

 The conclusions are justified and supported by the data, however, it would be beneficial to have more information about product development, marketing aspects and further development directions. The presented technologies can be considered as new techniques in the households, therefore reading about some forecasting of their future in the households would be really exciting as well.

 This article is scientifically valid in its current form, the above-listed recommendations refer to minor changes for improvement, but the raised issues are not hindering the approval of the report.

If applicable, is the statistical analysis and its interpretation appropriate?

Yes

Is the study design appropriate and is the work technically sound?

Yes

Is the work clearly and accurately presented and does it engage with the current literature?

Yes

Are the conclusions drawn adequately supported by the results?

Yes

Are sufficient details of methods and analysis provided to allow replication by others?

Partly

Are all the source data and materials underlying the results available?

Partly

Reviewer Expertise:

Consumer science, food science

We confirm that we have read this submission and believe that we have an appropriate level of expertise to confirm that it is of an acceptable scientific standard.

# Response

**Published:** 2022-02-05

**Authors:** Clara Talens

**Affiliations:** AZTI, Food Research, Basque Research and Technology Alliance (BRTA), Spain

The authors highly appreciate the valuable contribution of reviewers Gyula Kasza and Tekla Izsó. We have now created a new version incorporating the changes suggested by the reviewers. Please find below our responses to each comment in *italics*.

 Despite the fact that the experimental design and the interpretation of the used methods are adequate, the wide focus poses some challenges for the authors and the readers, as well. A flow chart or infographics indicating the sequence and interdependencies of the different research stages would provide significant support for better comprehension.

*Response: A figure has been added at the beginning of the article to explain the methodology.*

 Some minor additions and changes in the description of the methodology may improve the understanding of the study such as:

- The breakfast types of the selected countries should be edited in a table to indicate the differences and similarities among them.

*Response: The authors believe that the differences are already stated in the examples given. *

- The description of the sampling goals should be explained (representativity aspects also) in the Methodology section.

 
*Response: A figure has been added at the beginning of the article to explain the methodology.*

- A description of the pilot testings would improve the scientific quality.

*Response: Few details on the pilot testing have been added to the sentence.*

-  The "collaboration model" should be emphasized better in the introduction and research design parts.

*Response: A sentence about the collaboration model has been included.*

 The results are presented accurately, however, in some points clarification would be needed. Answering and checking the following questions in the text of the report and completing this section would be advantageous (but not essential):

-  In the "Special foods" section: Were "wheat-free" and "gluten-free" different claims in the study? Or does this refer to an open-ended question? Or is it just a translation bias?

*Response: It was indeed a translation bias, corrected to 'gluten-free'.*

-  An explanation of the "multi-channel tool" would be great.

*Response: An explanation of multi-channel tool has been added.* 

- The paper contains only a few references to other studies. I recommend identifying similar RDI projects from the food sector and comparing their findings (and the methodological approach) to the actual study

*Response: A new discussion section has been created.*

 It is understandable that the research data is available in part due to the business interests of the participating companies. Nevertheless, the findings are supported by the outcome of the research.

 The conclusions are justified and supported by the data, however, it would be beneficial to have more information about product development, marketing aspects and further development directions. The presented technologies can be considered as new techniques in the households, therefore reading about some forecasting of their future in the households would be really exciting as well.

*Response: The article itself means to be a forecast of the future of smart devices in households, this is included in the discussion and conclusion section in general terms.*

 This article is scientifically valid in its current form, the above-listed recommendations refer to minor changes for improvement, but the raised issues are not hindering the approval of the report.
